# Author Correction: Biocatalytic routes to stereo-divergent iridoids

**DOI:** 10.1038/s41467-022-33380-z

**Published:** 2022-09-27

**Authors:** Néstor J. Hernández Lozada, Benke Hong, Joshua C. Wood, Lorenzo Caputi, Jérôme Basquin, Ling Chuang, Maritta Kunert, Carlos E. Rodríguez López, Chloe Langley, Dongyan Zhao, C. Robin Buell, Benjamin R. Lichman, Sarah E. O’Connor

**Affiliations:** 1 Max-Planck Institute for Chemical Ecology, Department of Natural Product Biosynthesis, Hans-Knoll Strasse 8, 07745 Jena, Germany; 2 Center for Applied Genetic Technologies, University of Georgia, Athens, GA USA; 3 Max-Planck Institute for Biochemistry, Department of Structural Cell Biology, Am Klopferspitz 18, 82152 Martinsried, Germany; 4 University of York, Department of Biology, Centre for Agricultural Products, Wentworth Way, York, YO10 5DD UK

**Keywords:** Natural products, Secondary metabolism, Enzyme mechanisms, Biocatalysis, Enzymes

Correction to: *Nature Communications* 10.1038/s41467-022-32414-w, published online 11 August 2022

The original version of this Article contained an error in the first sentence of the “Results and Discussion” section, which incorrectly read ‘With a total of 19 wild-type NEPS sequences ranging from 64–96% sequence identity and two 3D NEPS structures to compare and contrast (Supplementary Figs. 1 and 2) we sought to understand the.’ The correct version adds ‘residues involved in the various catalytic activities observed.’ after ‘the’. This has been corrected in both the PDF and HTML versions of the Article.

