# Supplementary material for: Biocatalytic routes to stereo-divergent iridoids
Source: Nat Commun. 2022 Aug 11;13:4718. doi: 10.1038/s41467-022-32414-w (PMC9372074; doi:10.1038/s41467-022-32414-w)
Supplement: Supplementary file 1 — Supplementary Information [file 41467_2022_32414_MOESM1_ESM.pdf]

Supplementary Information for:

Biocatalytic routes to stereo-divergent iridoids

Néstor J. Hernández Lozada[a], Benke Hong[a], Joshua C. Wood[b], Lorenzo Caputi[a], Jerome Basquin[c], Maritta Kunert[a], Ling Chuang[a], Chloe Langley[a], Dongyan Zhao[b], C. Robin Buell[b], Benjamin R. Lichman[d], Sarah E. O'Connor[a,e]

[a] Max-Planck Institute for Chemical Ecology, Department of Natural Product Biosynthesis, Hans-Knoll Strasse 8, 07745, Jena, Germany.

[b] Michigan State University, Department of Plant Biology, East Lansing MI, USA

[c] Max-Planck Institute for Biochemistry, Department of Structural Cell Biology, Am Klopferspitz 18, 82152, Martinsried, Germany.

[d] University of York, Department of Biology, Centre for Agricultural Products, Wentworth Way, York, YO10 5DD, U.K.

[e] Corresponding author

List of Tables:

Supplementary Table 1. NsNEPS2 crystal structure data collection and refinement statistics

Primers used for cloning *N. sibirica* genes from cDNA.

Supplementary Table 2. Primers used for cloning *N. sibirica* genes from cDNA.

Supplementary Table 3. *Nepeta sibirica* and *Lamium album* genes cloned.

Supplementary Table 4: Summary of *7S* stereoselective enzymatic activities

Supplementary Table 5: Summary of *7R* stereoselective enzymatic activities

Supplementary Table 6: Primers used for generating mutants in this study.

List of Figures:

Supplementary Figure 1. Pairwise comparison of amino acid identities of NEPS sequences.

Supplementary Figure 2. Sequence alignment of all NEPS enzymes.

Supplementary Figure 3. NEPS catalytic tyrosine mutations to phenylalanine.

Supplementary Figure 4. NcNEPS3A residue 206 variations

Supplementary Figure 5. NcNEPS3A loop swaps

Supplementary Figure 6. Engineering 7*S*-*trans*-*cis* nepetalactol cyclization in NmNEPS1.

Supplementary Figure 7. Variations of the 155SATA residues in NmNEPS1

Supplementary Figure 8. Variations of the S199 residue in NmNEPS1-155SATA variant enzyme.

Supplementary Figure 9. NsNEPS1B mutagenesis.

Supplementary Figure 10. Chiral column nepetalactone standards MS data

Supplementary Figure 11. Influence of 8-oxoneral as a substrate to ISY in the resulting product profile.

Supplementary Figure 12. *N. sibirica* NEPS and MLPL assayed in combination with *Lamium album* LaISY.

Supplementary Figure 13. *N. mussinii, N. cataria* and *H. officinalis* selected NEPS and MLPL assays

Supplementary NMR Methods and Data

Supplementary Figure 14. Proton and Carbon NMR of 7*S*-*trans-trans* nepetalactone.

Supplementary Figure 15. Proton and Carbon NMR of 7*R*-*trans-trans* nepetalactone.

Supplementary Figure 16. Proton and Carbon NMR of 7*R*-*cis-cis* nepetalactone.

Supplementary Figure 17. Proton and Carbon NMR of 7*S*-*cis-cis* nepetalactone.

Supplementary Figure 18: Representative SDS-PAGE gel of proteins purified for Figures 2D and S6.

Supplementary Table 1. NsNEPS2 crystal structure data collection and refinement statistics.

	NsNEPS2*
**Wavelength**	1.00
**Resolution range**	49.54 - 1.85 (1.916 - 1.85)
**Space group**	P 21 21 21
**Unit cell**	68.753 106.68 142.876 90 90 90
**Total reflections**	1077784 (57590)
**Unique reflections**	88386 (7393)
**Multiplicity**	12.2 (7.8)
**Completeness (%)**	97.81 (82.82)
**Mean I/sigma(I)**	23.33 (2.05)
**Wilson B-factor**	36.43
**R-merge**	0.05726 (0.7775)
**R-meas**	0.05975 (0.8335)
**R-pim**	0.01681 (0.2918)
**CC1/2**	0.999 (0.798)
**CC***	1 (0.942)
**Reflections used in refinement**	88361 (7392)
**Reflections used for R-free**	4417 (369)
**R-work**	0.1718 (0.3073)
**R-free**	0.1974 (0.3428)
**CC(work)**	0.969 (0.870)
**CC(free)**	0.962 (0.803)
**Number of non-hydrogen atoms**	8195
**macromolecules**	7467
**ligands**	280
**solvent**	552
**Protein residues**	1031
**RMS(bonds)**	0.008
**RMS(angles)**	0.89
**Ramachandran favored (%)**	97.85
**Ramachandran allowed (%)**	2.15
**Ramachandran outliers (%)**	0.00
**Rotamer outliers (%)**	0.00
**Clashscore**	5.77
**Average B-factor**	39.2
**macromolecules**	38.89
**ligands**	36.35
**solvent**	44.31

Statistics for the highest-resolution shell are shown in parentheses.

Supplementary Table 2. Primers used for cloning *N. sibirica* genes from cDNA.

**NEPS**	**Primer FW**	**Primer RV**
NsNEPSL	AAGTTCTGTTTCAGGGCCCGGCGAACAATTTCCTCATGC	ATGGTCTAGAAAGCTTTATTTTGGAGGGGTGACG
NsNEPS1A	AAGTTCTGTTTCAGGGCCCGGCAAGCATTGTAAATCCGG	ATGGTCTAGAAAGCTTTATGTTGTTGAAGGTGCAACG
NsNEPS1B	AAGTTCTGTTTCAGGGCCCGGCAAGCATTGTAAATCCGG	ATGGTCTAGAAAGCTTTAGGATGAAGGAGCAAAGAATG
NsNEPS2	AAGTTCTGTTTCAGGGCCCGGGCCACAAGAAGAAGCTC	ATGGTCTAGAAAGCTTTATGAATGGGCGGCGAAT
NsNEPS4A	AAGTTCTGTTTCAGGGCCCGGCAAGCATTGTAAATCCGG	ATGGTCTAGAAAGCTTTATGTTGTTGAAGGTGCAACG
NsNEPS4B	AAGTTCTGTTTCAGGGCCCGGCAAGCATTGTAAATCCGG	ATGGTCTAGAAAGCTTTATGTTGTTGAAGGTGCAACG
**MLPL**	**Primer FW**	**Primer RV**
NsMLPL1	AAGTTCTGTTTCAGGGCCCGGCTTCCAAGCTTGAAGTGG	ATGGTCTAGAAAGCTTTATGCCTTGAGAACATAATCAT
NsMLPL2	AAGTTCTGTTTCAGGGCCCGGCTTCAAAGATTGAAGTAGAAAT	ATGGTCTAGAAAGCTTTATGCCTTGAGAACATAATCAT
NsMLPL3	AAGTTCTGTTTCAGGGCCCGGCTTCAAAACTTGAAGTAGAAAT	ATGGTCTAGAAAGCTTTATTCCTTGAGAAGATAATCATCCA
**ISY**	**Primer FW**	**Primer RV**
NsISY	AAGTTCTGTTTCAGGGCCCGAGCTGGTGGTGGGCTG	ATGGTCTAGAAAGCTTCAAGGAACAATCTTGTAAGCCTT
NsP5βR	AAGTTCTGTTTCAGGGCCCGAGCTGGTGGTGGGCTG	ATGGTCTAGAAAGCTTTAAGGAACAATCTTGAAAGCT

Supplementary Table 3: *Nepeta Sibirica* and *Lamium album* cloned genes

Gene	Sequence
NsNEPSL	ATGGCGAACAATTTCCTCATGCAATTGAAGAAGCTCGAAGGCAAAGTAGCCATTGTAACTGGCGGCGCCAGTG GCATCGGCGAGGCCACCGCCCGCCTCTTCGCGAATCGCGGCGCCCACGCCGTGGTTATAGCCGACATTCAGCA GGAGAAGGGCCGCTCCGTGGCGGAATCCATCGGGACGCAGCGGTCTAGCTACATCCACTGCGACGTCACCGAC GAGGAGCATGTTAAGTCTATGGTAGAACGGACCGCCGCCACCTACGGCCGCGTGGACATATTGTTCAGCAACG CCGGCATCGTGGCCAACTCCTCTCAAACCATCCTCGACCTCGACCTCGACCAGTACGATCGCGTCATGCGTGTC AACACGCGCGGCATGGCCGCGTGCGTGAAGCACGCAGCGCGTAAGATGGTGGAGCTGGGAACGAGAGGCGCT ATTATCTGCACCGGCAGTGCTGCGGCGGCGAAGGGGGCACCGACCGGGACGGACTATGTGATGTCGAAGCAC GCGGTGTTGGGGCTGGTGCGGTCGGCGAGCATTCAGCTTGGGGCCCACGGGATTAGGGTTAACTGTGTGTCGC CGAGTGGGGTGCCGACGCCGCTCAGCGAAAAGGTTATTTGTGGGACGGCGAGTGATGTGGAGAGTGCTTTTGG ACCGTTCACGAGCTTGAAAGGGGTGGCGCCGACGGCGGTACACGTGGCGGAGGCGGTGGCGTTTCTGGCGTCG GAGGAGGCCGCTTTCGTGACGGGGCATGATTTGCTGGTGGATGGTGGCCTGCTTTCTTTACCATTCGTCACCCC TCCAAAATAA
NsNEPS1A	ATGGCAAGCATTGTAAATCCGGTGCAGGTGATGAAGAAGAAGCTGGAAGGCAAAGTTGTGATAGTAACAGGC GGGGCGAGCGGCATCGGGGAGACGGCAGCGCGTGTGTTTGCGCAACATGGCGCGCGTGCAGTGGTGATCGCT GACATCCAATCTGAAGTTGGGAAGTCCGTGGCGGAGTCCATCGGGAAGCGGTGCAGCTACGTCCAGTGCGACG TCTCGGACGAGGAGCAGGTAAAGTCGATGATAGAATGGACGGCCAGCACGTACGGCGGGCTGGACGTGATGT TCTCCAATGTGGGCATCATGAGCAGCTCCGCTCAAACCGTGATGGACCTCGACCTTTCGGAGTACGATAAGGT GATGCGTGTGAACGCGCGCGGGACGGCCGCGTGCTTGAAGCAGGCGGCGCGTAAGATGGTAGAGCTGGGAAC GAGAGGCACTATTATCTGCACGACCAGCGTGGTGTCGTGCAGGGGCGGGCAAAGCCTGACGGACTATGTGATG TCGAAGCACGCGGTGTTGGGGCTGGTCCGGTCGGCGAGCATACAGCTGGGGGCCCACGGGATTAGGGTTAACT GCGTGTCGCCGTCGGTGGTGATCACGCCGCTCGCCCAAAGGATGGGGTTTTCCACGCCCGATGATTTCCATACT CATTTTGGCAACTTCACTAGCCTCAAAGGAGTCTGCCTCACCGCCGACCACGTCGCCCAAGCCGTCGCCTTTCT CGCTTCCGACGACGCCGCGTTCATCACCGGACATAATTTGGACGTCGATGGTGGACTGCTTTGTTTACCATTCG TTGCACCTTCAACAACATAA

NsNEPS1B	ATGGCAAGCATTGTAAATCCGGTGCAGGTGATGAAGAAGAAGCTGGAAGGCAAAGTTGTGATAGTAACGGGC<br>GGGGCGAGCGGCATCGGGCAGACGGCAGCGCGTGTGTTTGCGCAACATGGCGCGCGTGCAGTGGTGATCGCTG<br>ACATCCAATCTGAAGTTGGGAAGTCCGTGGCGGAGTCCATCGGGAAGCGGTGCAGCTACGTCCAGTGCGACGT<br>CTCGGACGAGGAGCAGGTAAAGTCGATGATAGAATGGACGGCCAGCACGTACGGCGGGCTGGACGTGATGTT<br>CTCCAATGTGGGCATCATGAGCAGTTCCGCTCAAACCGTAATGGACCTCAACCTTGGGGAGTTCGATAAGGTG<br>ATGCGTGTGAACGCGCGCGGGACGGCCGCGTGCTTGAAGCAGGCGGCGCGTAAGATGGTAGAGCTGGGAACG<br>AGAGGCACTATTATCTGCACGACCAGCGCGATGTCGTCCAGGGGCGGGCAAAGCATGACGGACTATGTGATGT<br>CGAAGCACGCGGTGTTGGGGCTGGTCCGGTCGGCGAGCATGCAGCTGGGGGCCCACGGGATTAGGGTTAACTG<br>CGTGTCGCCGTCGTTGGTGATCACGCCGCTCGCCCAAAGGATGGGGTTTTCCACGCCCGATGATTTCCATACTC<br>ATTTGGCAACTTCACTAGCCTCAAAGGAGTCTGCCTCACCGCCGACCACGTCGCCCAAGCCGTCGCCTTTCTC<br>GCTTCCGACGACGCCGCCTTCATCACTGGACATGATTTGGTCCTCGATGGTGGACTGCTTTGTTTACCATTCTTT<br>GCTCCTTCATCCTAA
NsNEPS2	ATGCACAAGAAGAAGCTCGAAGGCAAAGTAGCCATTGTAACCGGCGGCGCCAGCGGCATCGGCGAGACCGCC<br>GCCCGCATATTCGCCGACCACGGCGCGCGTGCGGTGGTGATCGCCGATATTCAGTCGGAATTGGGCCGGATGG<br>TAGCGGAATCCATTGGGGCGAAGCGGTGCAGCTACGTGCAATGCGACATCGCCGACGAGGAGCAGGTTAAGT<br>CCGCGGTAGAATGGACGGCCACCACCTACGGCGGCCTCGACGTGATGTTCTGCAACGCCGGCATCATGAGCCA<br>CTCTGACTCCGGACAGACGGTGATGGAGCTCGATATGTCAAAGTTCGACGAGGTGATGCGTGTGAACACGCGC<br>GGGACGGCAGCGTGCGTGAAGCAGGCGGCGCGTAAGATGGTGGAGCTGGGAACGAGGGGCGGCGCCATCATC<br>TGCACCAGCAGCCCGCTGGCGACGAGGGGCGGACACGTCGACACGGACTACGTGATGTCGAAGCACGCGGTG<br>TTGGGGCTGGTGCGGTCGGCCAGCATGCAGCTTGGGGCCCACGGGATTAGGGTTAACAGCGTGTCGCCGATGG<br>CCGTGCTAACGCCGCTCACCCGGAGGATGGGGCTTGCCACGCCCGCTGACGTCGAGAATGCCTTGGGGCGGTT<br>CACTAGCTTGAAAGGGGTGGCGCTCACGGCCGAGCACGTCGCTGAAGCGGCGGCCTTCCTGGCTTCCGATGAG<br>GCGGCTTTCATCACCGGCCATGATCTGGTGGTGGATGGCGGACTGCTTTGTTTACCATTCGCCGCCCATTCATA<br>A
NsNEPS4A	ATGGCAAGCATTGTAAATCCGGTGCAGGTGATGAAGAAGAAGCTGGAAGGCAAAGTTGTGATAGTAACAGGC<br>GGGGCGAGCGGCATCGGGGAGACGGCAGCGCGTGTGTTTGCGCAACATGGCGCGCGTGCAGTGGTGATCGCT<br>GACATCCAATCTGAAGTTGGGAAGTCCGTGGCGGAGTCCATCGGGAAGCGGTGCAGCTACGTCCAGTGCGACG<br>TCTCGGACGAGGAGCAGGTAAAGTCGATGATAGAATGGACGGCCAACACGTATGGCGGGCTGGACGTCATGTT<br>CTGCAATGCGGGCATCATTACCTACTCCCCTCAAACCATAATGCACCTCGACCTCTCGCAATTCGATAAGGTGA<br>TGCGTGTGAACGCACACGGGACGGCCGCGTGCGTGAAGCAGGCGGCGCGTAAGATGGTGGAGCTGGGAACGA<br>GAGGCACTATTATCTGCACGACTAGCGCGACAGCATCCAAGGGCGGACAAAACATGACGGACTATGCGATGTC<br>GAAGCACGCGGTGGTGGGGCTGGTCCGGTCAGCGAGCATGCAGCTGGGGGCCCACGGGATTAGGGTTAACTG<br>CGTGTCGCCCTCGGCGGTGCTCACGCCGCTCGCCCAAAGGATGGGGATTGCCACGCCTGATGATTTATATACTC<br>ATTTGGCAACTTCACTAGCCTCAAAGGAGTCTACCTCACCGCCGACCAAGTCGCCGAAGCCGTCACCTTTCTC<br>GCTTCCGACGACGCTGCTTTCATCACCGGACATAATTTGGACCTCGATGGTGGACTGCTTTGTTTACCATTCGTT<br>GCACCTTCAACAACATAA
NsNEPS4B	ATGGCAAGCATTGTAAATCCGGTGCAGGTGATGAAGAAGAAGCTTGAAGGCAAAGTTGTGGTAGTAACGGGC<br>GGGGCGAACGGCATCGGGGAGACGGCGGCGCGCGTGTTTGCGGAGCATGGCGCGCGTGCGGTGGTGATTGCT<br>GACATCCAATCTGAAGTTGGGCAGTCCGTGGCGGAGGCCATCGGGGAGGGGTGCAGCTACGTCCAGTGCGACA<br>TCTCGGACGAGGAGCAGGTTAAGTCGATGATAGAATGGACGGCCAACACGTATGGCGGGCTGGACGTCATGTT<br>CTGCAATGCGGGCATCATTACCTACTCCCCTCAAACCATAATGCACCTCGACCTCTCGCAATTCGATAAGGTGA<br>TGCGTGTGAACGCACACGGGACGGCCGCGTGCGTGAAGCAGGCGGCGCGTAAGATGGTGGAGCTGGGAACGA<br>GAGGCACTATTATCTGCACGACTAGCGCGACAGCATCCAAGGGCGGACAAAACATGACGGACTATGCGATGTC<br>GAAGCACGCGGTGGTGGGGCTGGTCCGGTCAGCGAGCATGCAGCTGGGGGCCCACGGGATTAGGGTTAACTG<br>CGTGTCGCCCTCGGCGGTGCTCACGCCGCTCGCCCAAAGGATGGGGATTGCCACGCCTGATGATTTATATACTC<br>ATTTGGCAACTTCACTAGCCTCAAAGGAGTCTACCTCACCGCCGACCAAGTCGCCGAAGCCGTCACCTTTCTC<br>GCTTCCGACGACGCTGCTTTCATCACCGGACATAATTTGGACCTCGATGGTGGACTGCTTTGTTTACCATTCGTT<br>GCACCTTCAACAACATAA
NsMLPL1	ATGGCTTCCAAGCTTGAAGTGGAGCTCGAGTTGAAATCTGATGTAGAAAAAATGTGGAAAAACTTTAAGGAAT<br>TTACAAAATTATTCCCCAAGGCTTTGCCACATCTTTACGAAGGGATTGCCGTTGCCGAGGGCGATGGGATATCC<br>GCCGGAACAATCTTCATAAGCACTCTTAAACCGACAGATCCGTCTAACCCCGTGGTTTCGATCAACAAGGAGA<br>GGATTGATTCTCTAGATGATGAAAAGAAAATACTGACTTATAGTTATATTGAAGGGGAAATCCTAAAAAGTTA<br>CAAGAATTTGAAGGGCACAGTCTCTATGAGCAGCAACAATGGTGATGGAACTATATTTAAATATGTAGTTGAA<br>TTTGACAAGGCAAATGCACAAGTGCCAGATCCTCTTTTATTCAAGGACTTTTTGGTAATGGTCTTCCAAGGTTTT<br>GATGATTATGTTCTCAACGCATAA
NsMLPL2	ATGGCTTCAAAACTTGAAGTAGAAATTGAGTTGAAAACTCATTCAGAAAATCTGTGGAAAAACCTGAAAGAAT<br>TCATCACTTTCTTCCCCAAAGCATTGCCAAATATGTACGAAAAGATCGATGTGATCGAAGGCGATGGAAGATC<br>AGTTGGATCTGTCTTTGTGTCTACTCTAAAGCCATCAGAGTTAAACCCTGTGGTTGAGGTCACAAAGGAGAGGA<br>TCGAACTGTTGGATGAAGAGAAGAAAATATTGAGTTACAGTTTCGTTGAGGGAGAAATTTTGAAAAATTACAA<br>GAATTTCAGGGCCATAATTCGTGTGAGCAGAAGCAAAAGTGATGGGACTATAGTTAATTATTTAGCTGAATTT<br>GAGAAGGCAAATGCAGAAGTCCCAAATCCTGATTTCTTCAAAGATTACGTTGCCAAACTTTTCCATGATGTGGA<br>TGATTATCTTCTCAAGGAATGA

NsMLPL3	ATGGCTTCAAAGATTGAAGTAGAAATTGAGTTGAAAACTCCTTCAGATAAACTGTGGAAAAACCTGAAAGAAT<br>TCGTTTTCTTCTTCCCCAAAGCTTTGCCACATATGTTCGAGAAGATTGATGTGATAGAAGGCGATGGAAGATCA<br>GTTGGATCTGTATTTGTGGCCACTGTTAAGCCATCAGAGTTATACCCGGTGGTTACCACAAAGGAGAGGATTGA<br>AATGGTTGATGAAAAAAATAAGATGATGAGTTACAGTTTTGTTGAGGGTGAAATGTTGAAAAATTACAAGAAT<br>TTCAAGGCCACAATGTGTGTGAGCAGCAACAAAAATGATGGGTCTATAATCAAATATACAGCTGAATTTGAGA<br>AGGCAAATGCAGTTCCAGATCCATATTTCGTTACGGATAATGCTGCTAAACTTTTACATGACGTGGATGATTAT<br>CTTCTCAAGGCATGA
NsISY	ATGAGCTGGTGGTGGGCTGGAGCTACTGGCGCTGCCAAGAAAAGAATAGATGAAGAGGAGTCACTCCTAAAC<br>CACCAATGCGTAGCTCTGATAGTCGGGGTGACCGGACTCATCGGCAACAGCCTGGCGGAGATCCTGCCGCTCT<br>CCGACACCCCCGGCGGCCCATGGAAGGTATACGGTGTGGCGCGCCGCCCCCGTCCCTCCTGGAACGAGGATCA<br>CCCCATCACCTACATCTCATGCGACGTAACCAACACAGCCGACGTGGAGGCCAAGCTATCCCCTCTCACCGAC<br>GTAACACACATCTTCTACGCCACGTGGACCAGCCGATCCACCGAGGAGGAGAACTGCGAAGCCAACGGGAAA<br>ATGCTGAAAAATGTGCTGGACGCAATGATCCCTAACTGCCCCAATTTGAAGCATATCTGCTTGCAGACCGGTA<br>GATTCCACTACGTTGCTTCGGTTGTGGACTGGAAGATTAACAGCCACGACACTCCGTTAACCGAGGATTTACCT<br>CGATTGAACACGAAGAATTTCTACTATACGCAAGAGGATATTCTGTTTGAGGAGGTTAAGAAGAAGGAGGGGC<br>TGACATGGTCCGTGCATCGGCCCGGGACTATCTTCGGGTTCTCACCGTATAGCATGATGAATTTGGTTGGGACA<br>CTGTGTGTTTATGCAGCTATATGTAAGCACGAGGGTGCGGTTCTGAGGTTTCCTGGGTGTAAAGGTGCGTGGGA<br>AGGATTCTCGGATTGCGCGGATGCAGATTTGATCGCGGAGCATGAGATATGGGCGGCTATGGATCCTTACGCG<br>AAGAACGAGGCGTACAATGTGAGCAACGGGGATGTTTTCAAGTGGAAGCATTTCTGGAAGGTGCTGGCGGAG<br>AAGTTTGGGGTGGAATGCGGGGAGTACGAGGAAGGGCGAGAGGTGAAGCTGCAGGAGGTGATGAAGGATAA<br>AGGTCCGGTGTGGGACGAGATCGTGAGGGCGAACGGGTTGTCGAGTACGAAGTTGGAGGATGTGGGGAAATG<br>GTGGTTTAGTGATACTATTCTGTGGAATGAGTGTAGGTTGGATACTATGAATAAGAGCAAGGAGCATGGGTTT<br>CTTGGGTTTAGGAATTCCAAGAATTGCTTTGGTTATTGGATTGATAAGGTGAAGGCTTACAAGATTGTTCCTTG<br>A
NsP5βR	ATGAGCTGGTGGTGGGCTGGAGCTATTGGCGCTGCCAAGAAAAGAATCGATGAAGATGAGGCACCGCGGAAC<br>TACGAGAGCGTAGCTCTGATAGTGGGGGTGACCGGAATCGTAGGCAACAGCCTGGCGGAGATTCTCCCGCTCT<br>CCGACACTCCCAGTGGCCCATGGAAGGTTTATGGGGTGGCTCGCCGCCCCCGTCCCTCCTGGAACGACGATCA<br>CCCCATTACCTACATCTCCTGCGATGTATTGGACTCCGTCGACGTGGAGGCGAAGCTATCCCCTCTCACCGATG<br>TAACACACATATTCTATGCCACATGGACCAAGAGATCCACGGAGAAGGAGAACTGCGAAGCTAATGGGAAAA<br>TGCTGAAAAACGTGCTGAATGCAATGATCCCTAATTGCCCCAATTTGAAGCATATCTGTTTGCAGACTGGTAGG<br>AAGCATTATGTTGGTGCATTTGAGAATTGGAAGATTAAAAGAAGTCACGATCCTCCGTTCACTGAGGATTTGCC<br>TCGATTGGATTCCCAGAATTTCTATTATACACAAGAGGACATTCTGTTTGAGGAGGTTCAGAAGAAGGAGGGC<br>TTGACATGGTCTGTGCATCGGCCTGGGAATATTTTCGGGTTCTCACCGTATAGCATGATGAATTTGGTTGGAAC<br>GCTGTGTGTTTATGCAGCTATCTGTAAGCACGAGGGTGCAGTTTTGAGGTTTCCTGGTTGTAAGGGTGCCTGGG<br>ATGGATACTCGGATTGCTCGGATGCAGACTTGATTGCAGAGCATCAGATATGGGCGGCCGTGGATCCTTATGC<br>GAAGAATGAGGCATTCAATGTGAGCAACGGCGATGTTTTCAAATGGAAGCATTTCTGGAAGGTGTTGGCCGAA<br>CAGTTTGGCGTGGAATGTGGGGAGTATGAGGAAGGGCAGGAAGTGAAGTTGCAGGATCTGATGAAGGATAAA<br>GGTCCGATCTGGGACAAAATCGTGAGGGAGAATGGGTTGTCGGCTACGAAATTGGAGGATGTTGGGACTTGGT<br>GGTTTAGTGACATTATTCTCGGGAATGAATGTTGGTTGGATACAATGAACAAAAGCAAGGAGCATGGATTTCT<br>TGGATTCAGGAATTCCAAGAATTCCTTCATTTCTTGGATTGACAAGGTGAAAGCTTTCAAGATTGTTCCTTAA
LaISY	ATGCCGACCGAAACGATCATGAGTTGGTGGTATAAACGCAGCATTGGTGACATTGAACAGAAGAAACTTCAGT<br>CCAATGGCCATGCACCGAGCTACAAATCGGTTGCGCTTATTGTGGGAGTTACGGGCATTGCGGGATCTGGCTTA<br>GCTGAAACACTGTCGCTGGGTGATACTCCAGGAGGCCCGTGGAAAGTGTATGGGGTTGCACGGCGTCCGTGTC<br>CAGAGTGGCTTACCACACTCCATGTCGACTATATCCAGTGTGACATTGCCAACACCGAAGAAACGAACTCCAA<br>GCTGAGTCCGTTGAAAGATATTACCCATGTATTCTACGTGAGTTGGACAGGGAGTGAAGATGTTGCGCTGAAC<br>ACGCTGATGTTCCGCAATATTCTCGACTCGGTGATCCCGAATGCCCCGAATCTGAAACATGTGGCTCTGCAAAC<br>CGGGATCAAATACTACTGGGGCAACATGGCCGAGATGGAAAGCACTAATCAGCCGCATGAATGCCCCTTCTAT<br>GAGAATTTACCACGTCTGAAACAGGAAAACTTCTACTACAATCTGGAAGATTTGGTATATGAAGCAGGTTTGG<br>GTCGCTCATCACTGACTTGGTCTGTGCACCGTCCTGCGCTGATTTTCGGGTTTTCTCCTTGTTCGATGATGAACG<br>CCGTGAGCACCATGTGCGTCTATGCTGCGATCTGCAAACATGAGAACAAACCCCTGGTCTATACCGGTACCGA<br>AGTCAGCTGGACTTGTCTGTGGGATGCGGTAGATAGCGATCTGTTAGCCGATCACTTTGTTTGGGCAGGTACCG<br>ATCCGAAAGCAAAGAACGAGGCGTTTAACGTCAACAATGGCGATGTCTTTAAATGGAAACACATGTGGAAAGT<br>GTTGGGCGAACAATTCGGTATTGAAAGCGTTGGCTATGAAGGCAAGGAGCCTGTTCTTCTGGAGGACCTGATG<br>AAAGACAAAGATGGTGTATGGGACGAAATCGTAAAGAAACACGATCTCGTCCCAACCAAACTCAAAGACATC<br>GCGGCCTTTTGGCTGGTTGATGTGGTGTTTCGCAACAAAGAGACGTTATGCTCAATGAACAAGAATAAAGAAT<br>TCGGCTTTCTGGGTTTTCGTGACACGACCAAATCCTTTGTGAATTGCGTGAAGAAAATGCGCGATTATCGCTTT<br>ATTCCGTAA

Supplementary Table 4: Summary of *7S* stereoselective enzymatic activities

Figure	Substrate	ISY	NEPS/MLPL	7S iridodials	7S-trans-trans nepetalactol	7S-cis-trans nepetalactol	7S-trans-cis nepetalactol*	7S-cis-cis nepetalactol	7S-trans-trans nepetalactone	7S-cis-trans nepetalactone	7S-trans-cis nepetalactone	7S-cis-cis nepetalactone
2A	8-oxogeranial	CrISY	N/A	+++	n.d.	+++	n.d.	n.d.	n.d.	n.d.	n.d.	n.d.
2A	8-oxogeranial	CrISY	NsNEPS2	tr.	n.d.	n.d.	n.d.	n.d.	n.d.	+++	n.d.	n.d.
2A	8-oxogeranial	CrISY	NsNEPS2-Y167F	tr.	n.d.	+++	n.d.	n.d.	n.d.	n.d.	n.d.	n.d.
2B	8-oxogeranial	CrISY	NmNEPS3	+	n.d.	+	n.d.	+++	n.d.	n.d.	n.d.	tr.
2B	8-oxogeranial	CrISY	NcNEPS3A	+	n.d.	+	n.d.	+++	n.d.	n.d.	n.d.	++
2B	8-oxogeranial	CrISY	NcNEPS3A-V206Q	+	n.d.	+	n.d.	+++	n.d.	n.d.	n.d.	tr.
2B	8-oxogeranial	CrISY	NmNEPS3-Q206V	+	n.d.	+	n.d.	+++	n.d.	n.d.	n.d.	++
2D	8-oxogeranial	CrISY	NmNEPS1	n.d.	n.d.	n.d.	n.d.	n.d.	n.d.	+++	tr.	tr.
2D	8-oxogeranial	CrISY	NmNEPS4	tr.	n.d.	n.d.	++	n.d.	n.d.	++	tr.	n.d.
2D	8-oxogeranial	CrISY	NmNEPS1 + NmNEPS4	n.d.	n.d.	n.d.	n.d.	n.d.	n.d.	++	+++	n.d.
2D	8-oxogeranial	CrISY	NmNEPS1-8 mutation graft	tr.	n.d.	n.d.	+	n.d.	n.d.	++	n.d.	n.d.
2D	8-oxogeranial	CrISY	NmNEPS1-154SATA-S198L	tr.	n.d.	n.d.	++	n.d.	n.d.	++	n.d.	n.d.
2D	8-oxogeranial	CrISY	NmNEPS1-154SATA	tr.	n.d.	n.d.	n.d.	n.d.	n.d.	+++	++	tr.
2D	8-oxogeranial	CrISY	NmNEPS1-154SVTA	tr.	n.d.	n.d.	n.d.	n.d.	n.d.	++	++	tr.
S6	8-oxogeranial	CrISY	NmNEPS1-S198L	tr.	n.d.	n.d.	n.d.	n.d.	n.d.	+++	n.d.	tr.
S6	8-oxogeranial	CrISY	NmNEPS1 with NmNEPS4 loop	tr.	n.d.	n.d.	tr.	n.d.	n.d.	+++	+	tr.
S6	8-oxogeranial	CrISY	NmNEPS1-S198L with NmNEPS4 loop	tr.	n.d.	n.d.	tr.	n.d.	n.d.	+++	n.d.	n.d.
3C	8-oxogeranial	NsISY	N/A	+++	n.d.	+++	n.d.	n.d.	n.d.	n.d.	n.d.	n.d.
3C	8-oxogeranial	NsISY	NsNEPSL	n.d.	n.d.	n.d.	n.d.	n.d.	tr.	+++	tr.	tr.
3C	8-oxogeranial	NsISY	NsNEPS2	tr.	n.d.	n.d.	n.d.	n.d.	n.d.	+++	n.d.	n.d.
3C	8-oxogeranial	NsISY	NsNEPS4A	+++	n.d.	+++	n.d.	n.d.	n.d.	n.d.	n.d.	n.d.
3C	8-oxogeranial	NsISY	NsNEPS4B	+++	n.d.	+++	n.d.	n.d.	n.d.	tr.	n.d.	n.d.
3C	8-oxogeranial	NsISY	NsNEPS1A	n.d.	n.d.	n.d.	n.d.	n.d.	n.d.	+++	tr.	tr.
3C	8-oxogeranial	NsISY	NsNEPS1B	n.d.	n.d.	n.d.	n.d.	n.d.	+	+++	+	tr.
3C	8-oxogeranial	NsISY	NsMLPL1	tr.	n.d.	+++	n.d.	n.d.	n.d.	n.d.	n.d.	n.d.
3C	8-oxogeranial	NsISY	NsMLPL2	+++	n.d.	+++	n.d.	n.d.	n.d.	n.d.	n.d.	n.d.
3C	8-oxogeranial	NsISY	NsMLPL3	+++	n.d.	++	n.d.	n.d.	n.d.	n.d.	n.d.	n.d.
3D	8-oxogeranial	NsP5βR	N/A	+++	n.d.	+++	n.d.	n.d.	n.d.	n.d.	n.d.	n.d.
3D	8-oxogeranial	NsISY	N/A	+++	n.d.	+++	n.d.	n.d.	n.d.	n.d.	n.d.	n.d.
3D	8-oxogeranial	CrISY	N/A	+++	n.d.	+++	n.d.	n.d.	n.d.	n.d.	n.d.	n.d.
3D	8-oxogeranial	LaISY	N/A	tr.	n.d.	n.d.	n.d.	n.d.	n.d.	n.d.	n.d.	n.d.

Figure	Substrate	ISY	NEPS/MLPL	7S iridodials	7S-trans-trans nepetalactol	7S-cis-trans nepetalactol	7S-trans-cis nepetalactol*	7S-cis-cis nepetalactol	7S-trans-trans nepetalactone	7S-cis-trans nepetalactone	7S-trans-cis nepetalactone	7S-cis-cis nepetalactone
4B	N/A	*N. sibirica* leaf tissue extract		**n.d.**	**n.d.**	**n.d.**	**n.d.**	**n.d.**	**n.d.**	**+**	**n.d.**	**n.d.**
4B	8-oxogeranial	CrISY	NsNEPS1A	**n.d.**	**n.d.**	**n.d.**	**n.d.**	**n.d.**	**n.d.**	**+++**	**+**	**n.d.**
4B	8-oxogeranial	CrISY	NsNEPS1B	**n.d.**	**n.d.**	**n.d.**	**n.d.**	**n.d.**	**+**	**+++**	**++**	**n.d.**
4B	8-oxogeranial	LaISY	NsNEPS1A	**n.d.**	**n.d.**	**n.d.**	**n.d.**	**n.d.**	**n.d.**	**n.d.**	**n.d.**	**n.d.**
4B	8-oxogeranial	LaISY	NsNEPS1B	**n.d.**	**n.d.**	**n.d.**	**n.d.**	**n.d.**	**n.d.**	**n.d.**	**n.d.**	**n.d.**
S3	8-oxogeranial	CrISY	N/A	**+++**	**n.d.**	**+++**	**n.d.**	**n.d.**	**n.d.**	**n.d.**	**n.d.**	**n.d.**
S3	8-oxogeranial	CrISY	NsNEPS2-Y163F	**tr.**	**n.d.**	**+++**	**n.d.**	**n.d.**	**n.d.**	**n.d.**	**n.d.**	**n.d.**
S3	8-oxogeranial	CrISY	NsNEPS1A-Y167F	**++**	**n.d.**	**+++**	**n.d.**	**n.d.**	**n.d.**	**n.d.**	**n.d.**	**n.d.**
S3	8-oxogeranial	CrISY	NsNEPS1B-Y164F	**++**	**n.d.**	**+**	**n.d.**	**n.d.**	**n.d.**	**++**	**n.d.**	**n.d.**
S3	8-oxogeranial	CrISY	NcNEPS3A-Y165F	**+++**	**n.d.**	**+++**	**n.d.**	**n.d.**	**n.d.**	**n.d.**	**n.d.**	**n.d.**
S3	8-oxogeranial	CrISY	NmNEPS4-Y168F	**+++**	**n.d.**	**+++**	**n.d.**	**n.d.**	**n.d.**	**n.d.**	**n.d.**	**n.d.**
S3	8-oxogeranial	CrISY	NmNEPS1-Y167F	**+**	**n.d.**	**n.d.**	**n.d.**	**n.d.**	**n.d.**	**++**	**n.d.**	**n.d.**
S4	8-oxogeranial	CrISY	N/A	**+++**	**n.d.**	**+++**	**n.d.**	**n.d.**	**n.d.**	**n.d.**	**n.d.**	**n.d.**
S4	8-oxogeranial	CrISY	NcNEPS3A	**n.d.**	**n.d.**	**n.d.**	**n.d.**	**++**	**n.d.**	**n.d.**	**n.d.**	**+++**
S4	8-oxogeranial	CrISY	NcNEPS3A-V206M	**n.d.**	**n.d.**	**n.d.**	**n.d.**	**+++**	**n.d.**	**n.d.**	**n.d.**	**+**
S4	8-oxogeranial	CrISY	NcNEPS3A-V206E	**++**	**n.d.**	**++**	**n.d.**	**++**	**n.d.**	**n.d.**	**n.d.**	**n.d.**
S4	8-oxogeranial	CrISY	NcNEPS3A-V206N	**tr.**	**n.d.**	**+**	**n.d.**	**+++**	**n.d.**	**n.d.**	**n.d.**	**tr.**
S4	8-oxogeranial	CrISY	NcNEPS3A-V206G	**tr.**	**n.d.**	**+**	**n.d.**	**+++**	**n.d.**	**n.d.**	**n.d.**	**tr.**
S4	8-oxogeranial	CrISY	NcNEPS3A-V206L	**tr.**	**n.d.**	**+**	**n.d.**	**+++**	**n.d.**	**n.d.**	**n.d.**	**tr.**
S4	8-oxogeranial	CrISY	NcNEPS3A-V206A	**tr.**	**n.d.**	**+**	**n.d.**	**+++**	**n.d.**	**n.d.**	**n.d.**	**+**
S4	8-oxogeranial	CrISY	NcNEPS3A-V206I	**tr.**	**n.d.**	**+**	**n.d.**	**+++**	**n.d.**	**n.d.**	**n.d.**	**+**
S5	8-oxogeranial	CrISY	N/A	**+++**	**n.d.**	**+++**	**n.d.**	**n.d.**	**n.d.**	**n.d.**	**n.d.**	**n.d.**
S5	8-oxogeranial	CrISY	NcNEPS3A	**n.d.**	**n.d.**	**n.d.**	**n.d.**	**++**	**n.d.**	**n.d.**	**n.d.**	**+++**
S5	8-oxogeranial	CrISY	NcNEPS3A with NmNEPS1 loop	**+**	**n.d.**	**+++**	**n.d.**	**n.d.**	**n.d.**	**+**	**n.d.**	**tr.**
S5	8-oxogeranial	CrISY	NcNEPS3A with NmNEPS4 loop	**++**	**n.d.**	**+++**	**n.d.**	**n.d.**	**n.d.**	**++**	**n.d.**	**+**
S5	8-oxogeranial	CrISY	NcNEPS3A with NmNEPS5 loop	**tr.**	**n.d.**	**n.d.**	**n.d.**	**n.d.**	**n.d.**	**+++**	**n.d.**	**tr.**
S7	8-oxogeranial	CrISY	NmNEPS1-154SATA	**tr.**	**n.d.**	**n.d.**	**n.d.**	**n.d.**	**n.d.**	**+++**	**++**	**tr.**
S7	8-oxogeranial	CrISY	NmNEPS1-154SATS	**tr.**	**n.d.**	**n.d.**	**n.d.**	**n.d.**	**n.d.**	**+++**	**+**	**tr.**
S7	8-oxogeranial	CrISY	NmNEPS1-154SSTA	**tr.**	**n.d.**	**n.d.**	**tr.**	**n.d.**	**n.d.**	**+++**	**+++**	**tr.**
S7	8-oxogeranial	CrISY	NmNEPS1-154SALA	**tr.**	**n.d.**	**n.d.**	**n.d.**	**n.d.**	**n.d.**	**+++**	**+**	**tr.**
S7	8-oxogeranial	CrISY	NmNEPS1-154AATA	**tr.**	**n.d.**	**n.d.**	**+++**	**n.d.**	**n.d.**	**++**	**+**	**n.d.**
S7	8-oxogeranial	CrISY	NmNEPS1-154SATG	**tr.**	**n.d.**	**n.d.**	**n.d.**	**n.d.**	**n.d.**	**+++**	**++**	**tr.**

Figure	Substrate	ISY	NEPS/MLPL	7S iridodials	7S-trans-trans nepetalactol	7S-cis-trans nepetalactol	7S-trans-cis nepetalactol*	7S-cis-cis nepetalactol	7S-trans-trans nepetalactone	7S-cis-trans nepetalactone	7S-trans-cis nepetalactone	7S-cis-cis nepetalactone
S7	8-oxogeranial	CrISY	NmNEPS1-154SGTG	tr.	n.d.	n.d.	n.d.	n.d.	n.d.	+++	+	tr.
S7	8-oxogeranial	CrISY	NmNEPS1-154SVTA	tr.	n.d.	n.d.	n.d.	n.d.	n.d.	+++	+++	tr.
S7	8-oxogeranial	CrISY	NmNEPS1-154TASA	tr.	n.d.	n.d.	n.d.	n.d.	n.d.	+++	++	tr.
S7	8-oxogeranial	CrISY	NmNEPS1-154SAGA	tr.	n.d.	n.d.	n.d.	n.d.	n.d.	+++	tr.	tr.
S7	8-oxogeranial	CrISY	NmNEPS1-154SPTA	tr.	n.d.	n.d.	n.d.	n.d.	n.d.	+++	+++	tr.
S8	8-oxogeranial	CrISY	NmNEPS1-154SATA-S198M	+	n.d.	n.d.	n.d.	n.d.	n.d.	+++	n.d.	n.d.
S8	8-oxogeranial	CrISY	NmNEPS1-154SATA-S198L	tr.	n.d.	n.d.	+++	n.d.	n.d.	+++	n.d.	n.d.
S8	8-oxogeranial	CrISY	NmNEPS1-154SATA-S198P	+++	n.d.	+++	n.d.	n.d.	n.d.	n.d.	n.d.	n.d.
S8	8-oxogeranial	CrISY	NmNEPS1-154SATA-S198V	+++	n.d.	tr	+	n.d.	n.d.	+++	n.d.	n.d.
S8	8-oxogeranial	CrISY	NmNEPS1-154SATA-S198G	tr.	n.d.	n.d.	n.d.	n.d.	n.d.	+++	++	tr.
S8	8-oxogeranial	CrISY	NmNEPS1-154SATA-S198A	+	n.d.	n.d.	tr.	n.d.	n.d.	+++	+	tr.
S8	8-oxogeranial	CrISY	NmNEPS1-154SATA-S198T	+	n.d.	n.d.	n.d.	n.d.	n.d.	+++	++	tr.
S8	8-oxogeranial	CrISY	NmNEPS1-154SATA-S198C	+	n.d.	tr	tr.	n.d.	n.d.	+++	tr.	n.d.
S9	8-oxogeranial	CrISY	N/A	+++	n.d.	+++	n.d.	n.d.	n.d.	n.d.	n.d.	n.d.
S9	8-oxogeranial	CrISY	NsNEPS1B	n.d.	n.d.	n.d.	n.d.	n.d.	+	+++	+	tr.
S9	8-oxogeranial	CrISY	NsNEPS1B-151GAMS	tr.	n.d.	+++	n.d.	n.d.	n.d.	++	++	tr.
S9	8-oxogeranial	CrISY	NsNEPS1B-151SATS	n.d.	n.d.	n.d.	n.d.	n.d.	+	+++	++	tr.
S9	8-oxogeranial	CrISY	NsNEPS1B-151SATA	+++	n.d.	+++	n.d.	n.d.	n.d.	n.d.	n.d.	n.d.
S9	8-oxogeranial	CrISY	NsNEPS1B-S195L	+	n.d.	n.d.	n.d.	n.d.	n.d.	+++	+	tr.
S9	8-oxogeranial	CrISY	NsNEPS1B-151GSSA	tr.	n.d.	n.d.	n.d.	n.d.	n.d.	+++	++	tr.
S9	8-oxogeranial	CrISY	NsNEPS1B-151AAMS	+	n.d.	+++	n.d.	n.d.	n.d.	++	++	tr.
S9	8-oxogeranial	CrISY	NsNEPS1B-151ASTA	+	n.d.	n.d.	n.d.	n.d.	n.d.	+++	++	tr.
S9	8-oxogeranial	CrISY	NsNEPS1B-151ASMA	+	n.d.	+++	n.d.	n.d.	n.d.	++	++	tr.
S11	8-oxogeranial	CrISY	N/A	+++	n.d.	+++	n.d.	n.d.	n.d.	n.d.	n.d.	n.d.

Figure	Substrate	ISY	NEPS/MLPL	7S iridodials	7S-trans-trans nepetalactol	7S-cis-trans nepetalactol	7S-trans-cis nepetalactol*	7S-cis-cis nepetalactol	7S-trans-trans nepetalactone	7S-cis-trans nepetalactone	7S-trans-cis nepetalactone	7S-cis-cis nepetalactone
S11	8-oxogeranial	LaISY	N/A	**tr.**	**n.d.**	**n.d.**	**n.d.**	**n.d.**	**n.d.**	**n.d.**	**n.d.**	**n.d.**
S11	8-oxogeranial	NsISY	N/A	**+++**	**n.d.**	**+++**	**n.d.**	**n.d.**	**n.d.**	**n.d.**	**n.d.**	**n.d.**
S11	8-oxogeranial	NsP5βR	N/A	**+++**	**n.d.**	**+++**	**n.d.**	**n.d.**	**n.d.**	**n.d.**	**n.d.**	**n.d.**
S11	8-oxoneral	CrISY	N/A	**+++**	**n.d.**	**+++**	**n.d.**	**n.d.**	**n.d.**	**n.d.**	**n.d.**	**n.d.**
S11	8-oxoneral	LaISY	N/A	**++**	**n.d.**	**++**	**n.d.**	**n.d.**	**n.d.**	**n.d.**	**n.d.**	**n.d.**
S11	8-oxoneral	NsISY	N/A	**+++**	**n.d.**	**+++**	**n.d.**	**n.d.**	**n.d.**	**n.d.**	**n.d.**	**n.d.**
S11	8-oxoneral	NsP5βR	N/A	**+++**	**n.d.**	**+++**	**n.d.**	**n.d.**	**n.d.**	**n.d.**	**n.d.**	**n.d.**
S13	8-oxogeranial	CrISY	N/A	**+++**	**n.d.**	**+++**	**n.d.**	**n.d.**	**n.d.**	**n.d.**	**n.d.**	**n.d.**
S13	8-oxogeranial	CrISY	NcMLPLA	**n.d.**	**n.d.**	**+++**	**n.d.**	**n.d.**	**n.d.**	**n.d.**	**n.d.**	**n.d.**
S13	8-oxogeranial	CrISY	NcMLPLB	**n.d.**	**n.d.**	**+++**	**n.d.**	**n.d.**	**n.d.**	**n.d.**	**n.d.**	**n.d.**
S13	8-oxogeranial	CrISY	NcNEPS2	**n.d.**	**n.d.**	**+++**	**n.d.**	**n.d.**	**n.d.**	**n.d.**	**n.d.**	**n.d.**
S13	8-oxogeranial	CrISY	NcNEPS5	**n.d.**	**n.d.**	**n.d.**	**n.d.**	**n.d.**	**tr.**	**+++**	**tr.**	**n.d.**
S13	8-oxogeranial	CrISY	NmNEPSL1	**n.d.**	**n.d.**	**n.d.**	**n.d.**	**n.d.**	**n.d.**	**+++**	**n.d.**	**n.d.**
S13	8-oxogeranial	CrISY	NmNEPSL2	**n.d.**	**n.d.**	**n.d.**	**n.d.**	**n.d.**	**n.d.**	**+++**	**n.d.**	**n.d.**
S13	8-oxogeranial	CrISY	NmNEPS2	**n.d.**	**n.d.**	**+++**	**n.d.**	**n.d.**	**n.d.**	**+++**	**n.d.**	**n.d.**
S13	8-oxogeranial	CrISY	NmNEPS5	**n.d.**	**n.d.**	**n.d.**	**n.d.**	**n.d.**	**tr.**	**+++**	**tr.**	**n.d.**
S13	8-oxogeranial	CrISY	NmMLPL1	**n.d.**	**n.d.**	**+++**	**n.d.**	**n.d.**	**n.d.**	**n.d.**	**n.d.**	**n.d.**
S13	8-oxogeranial	CrISY	NmMLPL2	**n.d.**	**n.d.**	**+++**	**n.d.**	**n.d.**	**n.d.**	**n.d.**	**n.d.**	**n.d.**
S13	8-oxogeranial	CrISY	NmMLPL3	**+++**	**n.d.**	**++**	**n.d.**	**n.d.**	**n.d.**	**n.d.**	**n.d.**	**n.d.**
S13	8-oxogeranial	CrISY	NcMLPL4	**+**	**n.d.**	**+++**	**n.d.**	**n.d.**	**n.d.**	**n.d.**	**n.d.**	**n.d.**
S13	8-oxogeranial	CrISY	HoNEPSLB	**n.d.**	**n.d.**	**n.d.**	**n.d.**	**n.d.**	**tr.**	**+++**	**tr.**	**n.d.**
S13	8-oxogeranial	CrISY	HoNEPSLA	**n.d.**	**n.d.**	**n.d.**	**n.d.**	**n.d.**	**tr.**	**+++**	**tr.**	**n.d.**

Note: The prescence of a particular chemical was graded in the following relative scale: not detected (n.d.), traces (tr.) and three levels of detection beyond traces, (+, ++, and +++), where each "+" sign indicates increasing amounts.

Supplementary Table 5: Summary of *7R* stereoselective enzymatic activities

Figure	Substrate	ISY	NEPS/MLPL	7R iridodials	7R-trans-trans nepetalactol	7R-cis-trans nepetalactol	7R-trans-cis nepetalactol	7R-cis-cis nepetalactol	7R-trans-trans nepetalactone	7R-cis-trans nepetalactone	7R-trans-cis nepetalactone	7R-cis-cis nepetalactone
S12	8-oxogeranial	LaISY	N/A	+++	n.d.	+++	n.d.	n.d.	n.d.	n.d.	n.d.	n.d.
S12	8-oxogeranial	LaISY	NsNEPSL	n.d.	n.d.	n.d.	n.d.	n.d.	+	+++	n.d.	n.d.
S12	8-oxogeranial	LaISY	NsNEPS2	n.d.	n.d.	n.d.	n.d.	n.d.	n.d.	+++	n.d.	n.d.
S12	8-oxogeranial	LaISY	NsNEPS4A	+++	n.d.	+++	n.d.	n.d.	n.d.	n.d.	n.d.	n.d.
S12	8-oxogeranial	LaISY	NsNEPS4B	+++	n.d.	+++	n.d.	n.d.	n.d.	n.d.	n.d.	n.d.
S12	8-oxogeranial	LaISY	NsNEPS1A	n.d.	n.d.	n.d.	n.d.	n.d.	++	+++	n.d.	n.d.
S12	8-oxogeranial	LaISY	NsNEPS1B	n.d.	n.d.	n.d.	n.d.	n.d.	+++	+++	n.d.	n.d.
S12	8-oxogeranial	LaISY	NsMLPL1	+++	n.d.	++	n.d.	n.d.	n.d.	n.d.	n.d.	n.d.
S12	8-oxogeranial	LaISY	NsMLPL2	+++	n.d.	+	n.d.	n.d.	n.d.	n.d.	n.d.	n.d.
S12	8-oxogeranial	LaISY	NsMLPL3	+++	n.d.	+	n.d.	n.d.	n.d.	n.d.	n.d.	n.d.
3D	8-oxogeranial	NsP5βR	N/A	n.d.	n.d.	n.d.	n.d.	n.d.	n.d.	n.d.	n.d.	n.d.
3D	8-oxogeranial	NsISY	N/A	n.d.	n.d.	n.d.	n.d.	n.d.	n.d.	n.d.	n.d.	n.d.
3D	8-oxogeranial	CrISY	N/A	n.d.	n.d.	n.d.	n.d.	n.d.	n.d.	n.d.	n.d.	n.d.
3D	8-oxogeranial	LaISY	N/A	+++	n.d.	+++	n.d.	n.d.	n.d.	n.d.	n.d.	n.d.
4B	N/A	*N. sibirica* leaf tissue extract		n.d.	n.d.	n.d.	n.d.	n.d.	+++	+	n.d.	n.d.
4B	8-oxogeranial	CrISY	NsNEPS1A	n.d.	n.d.	n.d.	n.d.	n.d.	n.d.	n.d.	n.d.	n.d.
4B	8-oxogeranial	CrISY	NsNEPS1B	n.d.	n.d.	n.d.	n.d.	n.d.	n.d.	n.d.	n.d.	n.d.
4B	8-oxogeranial	LaISY	NsNEPS1A	n.d.	n.d.	n.d.	n.d.	n.d.	++	++	n.d.	n.d.
4B	8-oxogeranial	LaISY	NsNEPS1B	n.d.	n.d.	n.d.	n.d.	n.d.	+++	++	n.d.	n.d.
5B	8-oxogeranial	LaISY	N/A	+++	n.d.	+++	n.d.	n.d.	n.d.	n.d.	n.d.	n.d.
5B	8-oxogeranial	LaISY	NmNEPS3-Q206V	+	n.d.	+++	n.d.	n.d.	n.d.	n.d.	n.d.	n.d.
5B	8-oxogeranial	LaISY	NsNEPS2-Y167F	n.d.	n.d.	+++	n.d.	n.d.	n.d.	n.d.	n.d.	n.d.
5B	8-oxogeranial	LaISY	NsNEPS2	n.d.	n.d.	n.d.	n.d.	n.d.	n.d.	+++	n.d.	n.d.
5B	8-oxogeranial	LaISY	NsMLPL1 + NsNEPS1B	n.d.	n.d.	n.d.	n.d.	n.d.	++	+++	n.d.	+
5B	8-oxogeranial	LaISY	NmNEPS1-154SVTA	n.d.	n.d.	n.d.	n.d.	n.d.	+++	+	n.d.	n.d.
S9	8-oxogeranial	LaISY	N/A	+++	n.d.	+++	n.d.	n.d.	n.d.	n.d.	n.d.	n.d.
S9	8-oxogeranial	LaISY	NsNEPS1B	n.d.	n.d.	n.d.	n.d.	n.d.	++	+++	n.d.	n.d.
S9	8-oxogeranial	LaISY	NsNEPS1B-151GAMS	++	n.d.	n.d.	n.d.	n.d.	+	+++	n.d.	n.d.
S9	8-oxogeranial	LaISY	NsNEPS1B-151SATS	n.d.	n.d.	n.d.	n.d.	n.d.	+++	+++	n.d.	n.d.
S9	8-oxogeranial	LaISY	NsNEPS1B-151SATA	++	n.d.	+++	n.d.	n.d.	n.d.	n.d.	n.d.	n.d.
S9	8-oxogeranial	LaISY	NsNEPS1B-S195L	n.d.	n.d.	n.d.	n.d.	n.d.	+	+++	n.d.	n.d.

Figure	Substrate	ISY	NEPS/MLPL	7R iridodials	7R-trans-trans nepetalactol	7R-cis-trans nepetalactol	7R-trans-cis nepetalactol	7R-cis-cis nepetalactol	7R-trans-trans nepetalactone	7R-cis-trans nepetalactone	7R-trans-cis nepetalactone	7R-cis-cis nepetalactone
S9	8-oxogeranial	LaISY	NsNEPS1B-151GSSA	+	n.d.	n.d.	n.d.	n.d.	tr	+++	n.d.	n.d.
S9	8-oxogeranial	LaISY	NsNEPS1B-151AAMS	++	n.d.	n.d.	n.d.	n.d.	+	+++	n.d.	n.d.
S9	8-oxogeranial	LaISY	NsNEPS1B-151ASTA	++	n.d.	n.d.	n.d.	n.d.	+	+++	n.d.	n.d.
S9	8-oxogeranial	LaISY	NsNEPS1B-151ASMA	+++	n.d.	n.d.	n.d.	n.d.	+	+++	n.d.	n.d.
S11	8-oxogeranial	CrISY	N/A	n.d.	n.d.	n.d.	n.d.	n.d.	n.d.	n.d.	n.d.	n.d.
S11	8-oxogeranial	LaISY	N/A	+++	n.d.	+++	n.d.	n.d.	n.d.	n.d.	n.d.	n.d.
S11	8-oxogeranial	NsISY	N/A	n.d.	n.d.	n.d.	n.d.	n.d.	n.d.	n.d.	n.d.	n.d.
S11	8-oxogeranial	NsP5βR	N/A	n.d.	n.d.	n.d.	n.d.	n.d.	n.d.	n.d.	n.d.	n.d.
S11	8-oxoneral	CrISY	N/A	n.d.	n.d.	n.d.	n.d.	n.d.	n.d.	n.d.	n.d.	n.d.
S11	8-oxoneral	LaISY	N/A	+++	n.d.	+++	n.d.	n.d.	n.d.	n.d.	n.d.	n.d.
S11	8-oxoneral	NsISY	N/A	n.d.	n.d.	n.d.	n.d.	n.d.	n.d.	n.d.	n.d.	n.d.
S11	8-oxoneral	NsP5βR	N/A	n.d.	n.d.	n.d.	n.d.	n.d.	n.d.	n.d.	n.d.	n.d.
S13	8-oxogeranial	LaISY	N/A	+++	n.d.	+++	n.d.	n.d.	n.d.	n.d.	n.d.	n.d.
S13	8-oxogeranial	LaISY	NcMLPLA	++	n.d.	++	n.d.	n.d.	n.d.	n.d.	n.d.	n.d.
S13	8-oxogeranial	LaISY	NcMLPLB	++	n.d.	++	n.d.	n.d.	n.d.	n.d.	n.d.	n.d.
S13	8-oxogeranial	LaISY	NcNEPS2	n.d.	n.d.	+++	n.d.	n.d.	n.d.	n.d.	n.d.	n.d.
S13	8-oxogeranial	LaISY	NcNEPS5	n.d.	n.d.	n.d.	n.d.	n.d.	+++	++	n.d.	n.d.
S13	8-oxogeranial	LaISY	NmNEPSL1	n.d.	n.d.	n.d.	n.d.	n.d.	n.d.	+++	n.d.	n.d.
S13	8-oxogeranial	LaISY	NmNEPSL2	n.d.	n.d.	n.d.	n.d.	n.d.	n.d.	+++	n.d.	n.d.
S13	8-oxogeranial	LaISY	NmNEPS2	n.d.	n.d.	+++	n.d.	n.d.	n.d.	+	n.d.	n.d.
S13	8-oxogeranial	LaISY	NmNEPS5	n.d.	n.d.	n.d.	n.d.	n.d.	+++	++	n.d.	n.d.
S13	8-oxogeranial	LaISY	NmMLPL1	+++	n.d.	+	n.d.	n.d.	n.d.	n.d.	n.d.	n.d.
S13	8-oxogeranial	LaISY	NmMLPL2	+++	n.d.	+	n.d.	n.d.	n.d.	n.d.	n.d.	n.d.
S13	8-oxogeranial	LaISY	NmMLPL3	+++	n.d.	tr.	n.d.	n.d.	n.d.	n.d.	n.d.	n.d.
S13	8-oxogeranial	LaISY	NcMLPL4	+	n.d.	+++	n.d.	n.d.	n.d.	n.d.	n.d.	n.d.
S13	8-oxogeranial	LaISY	HoNEPSLB	n.d.	n.d.	n.d.	n.d.	n.d.	n.d.	+++	n.d.	n.d.
S13	8-oxogeranial	LaISY	HoNEPSLA	n.d.	n.d.	n.d.	n.d.	n.d.	n.d.	+++	n.d.	n.d.

Note: The presence of a particular chemical was graded in the following relative scale: not detected (n.d.), traces (tr.) and three levels of detection beyond traces, (+, ++, and +++), where each "+" sign indicates increasing amounts.

Supplementary Table 6: Primers used for generating mutants in this study.

figure number	Gene description	FW primer	RV primer
2	*NsNEPS2-Y163F*	CGGTCATGTAGACACTGATTTTGTTATGTCC AAACACGC	AATCAGTGTCTACATGACC
2	*NcNEPS3A_M2_SG V206Q*	GTGGTGACGCCACTCACCCGGAACCAGGGG ATTTCGTCGCCGGCTGATGTACAGAATGTT	CGGGTGAGTGGCGTCACCACGGCCATCGGCG ACACGCTGTTAA
2	*NmNEPS3_M2_SG Q206V*	GCCGTGGCGACGCCGCTCACCCGGAACGTT GGCATTTCGACGCCGGATGATGTACAGAAA	GGTGAGCGGCGTCGCCACGGCCATCGGCGAC ACGCTGTTAACCCT
3	*NmNEPS1 154SATA loop*	ACCACCTCTGCTACAGCAAGCCGTGGCGGG CAAAGTATGACCGATTATGCGATGAGC	CTTGCTGTAGCAGAGGTGGTGCAAATAATGG TACCACG
3	*NmNEPS1 154SATA loop + S198L*	GCTGGCGCAGCGTATGG	CCATACGCTGCGCCAGCGGGGTCAGCACCAC TAATGGCGTCACGCAGTTAA
3	*SVTA loop*	ACCACCTCTGTTACAGCAAGCCGTGGCGGG CAAAGTATGACCGATTATGCGATGAGC	CTTGCTGTAACAGAGGTGGTGCAAATAATGG TACCACG
S3	*NcNEPS3A-Y163F*	CATAACGTTACAGATTTTGTAATGTCCAAAC ATGCGGT	TACAAAATCTGTAACGTTATGCG
S3	*NmNEPS4-Y163F*	CAAAATATGACTGACTTTGCGATGAGTAAG CACGCC	TCGCAAAGTCAGTCATATTTTGGC
S3	*NmNEPS1-Y163F*	CAAAGTATGACCGATTTTGCGATGAGCAAA CATGC	TCGCAAAATCGGTCATACTTTGCC
S3	*NsNEPS1A-Y163F*	AGAGCCTGACCGACTTTGTGATGAGCAAGC ATG	ATCACAAAGTCGGTCAGGCTCTGTC
S3	*NsNEPS1B-Y163F*	CAAAGCATGACGGACTTTGTGATGTCGAAG CACG	TCACAAAGTCCGTCATGCTTTGCC
S4	*NcNEPS3A-V206M*	GATCTCCAGTCCAGCTGAT	TCAGCTGGACTGGAGATCCCCATATTGCGAG TAAGAGGAGTTAC
S4	*NcNEPS3A-V206E*	GATCTCCAGTCCAGCTGAT	TCAGCTGGACTGGAGATCCCTTCATTGCGAGT AAGAGGAGTTAC
S4	*NcNEPS3A-V206N*	GATCTCCAGTCCAGCTGAT	TCAGCTGGACTGGAGATCCCGTTATTGCGAG TAAGAGGAGTTAC
S4	*NcNEPS3A-V206G*	GATCTCCAGTCCAGCTGAT	ATCAGCTGGACTGGAGATCCCACCATTGCGA GTAAGAGGAGTTA
S4	*NcNEPS3A-V206L*	GATCTCCAGTCCAGCTGAT	ATCAGCTGGACTGGAGATCCCTAAATTGCGA GTAAGAGGAGTTA
S4	*NcNEPS3A-V206A*	GATCTCCAGTCCAGCTGAT	ATCAGCTGGACTGGAGATCCCAGCATTGCGA GTAAGAGGAGTTA
S4	*NcNEPS3A-V206I*	GATCTCCAGTCCAGCTGAT	ATCAGCTGGACTGGAGATCCCAATATTGCGA GTAAGAGGAGTTA
S5	*NcNEPS3A_NmNEPS 1 150-162 loop*	ACTACACCCCTGTCGAGCCGTGGTGGGCAAT CTATGACAGATTACGTAATGTCCAAACAT	GCTCGACAGGGGTGTAGTTGTGCAGATGATG CTACCTCCA
S5	*NcNEPS3A_NmNEPS 4 150-162 loop*	ACGTCGGCCACGGCAAGCAAGGGCGGCCAA AACATGACAGATTACGTAATGTCCAAACAT	GCTTGCCGTGGCCGACGTGGTGCAGATGATG CTACCTCCA
S5	*NcNEPS3A_NmNEPS 5 150-162 loop*	ACAAGCCCGGCAAGCACTATGGGCGGCCAC AATCTGACAGATTACGTAATGTCCAAACAT	AGTGCTTGCCGGGCTTGTCGTGCAGATGATG CTACCTCCA
S6	*NmNEPS1_NmNEPS4 150-162 loop*	ACCACGTCGGCCACGGCAAGCAAGGGCGGC CAAAACATGACCGATTATGCGATGAGC	CTTGCCGTGGCCGACGTGGTGCAAATAATGG TACCACG
S6	*NmNEPS1-S198L*	GCTGGCGCAGCGTATGG	CCATACGCTGCGCCAGCGGGGTCAGCACCAC TAATGGCGTCACGCAGTTAA
S6	*NmNEPS1-S198L_NmNEPS4 150-162 loop*	ACCACGTCGGCCACGGCAAGCAAGGGCGGC CAAAACATGACCGATTATGCGATGAGC	CTTGCCGTGGCCGACGTGGTGCAAATAATGG TACCACG
S7	*NmNEPS1-154SATS*	ACCACCTCTGCTACATCTAGCCGTGGCGGGC AAAGTATGACCGATTATGCGATGAGC	CTAGATGTAGCAGAGGTGGTGCAAATAATGG TACCACG
S7	*NmNEPS1-154SSTA*	ACCACCTCTTCAACAGCAAGCCGTGGCGGG CAAAGTATGACCGATTATGCGATGAGC	CTTGCTGTTGAAGAGGTGGTGCAAATAATGG TACCACG
S7	*NmNEPS1-154SALA*	ACCACCTCTGCTTTAGCAAGCCGTGGCGGGC AAAGTATGACCGATTATGCGATGAGC	CTTGCTAAAGCAGAGGTGGTGCAAATAATGG TACCACG
S7	*NmNEPS1-154AATA*	ACCACCGCAGCTACAGCAAGCCGTGGCGGG CAAAGTATGACCGATTATGCGATGAGC	CTTGCTGTAGCTGCGGTGGTGCAAATAATGG TACCACG
S7	*NmNEPS1-154SATG*	ACCACCTCTGCTACAGGTAGCCGTGGCGGG CAAAGTATGACCGATTATGCGATGAGC	CTACCTGTAGCAGAGGTGGTGCAAATAATGG TACCACG
S7	*NmNEPS1-154SGTG*	ACCACCTCTGGTACAGGTAGCCGTGGCGGG CAAAGTATGACCGATTATGCGATGAGC	CTACCTGTACCAGAGGTGGTGCAAATAATGG TACCACG
S7	*NmNEPS1-154SVTA*	ACCACCTCTGTTACAGCAAGCCGTGGCGGG CAAAGTATGACCGATTATGCGATGAGC	CTTGCTGTAACAGAGGTGGTGCAAATAATGG TACCACG
S7	*NmNEPS1-154TASA*	ACCACCACAGCTTCTGCAAGCCGTGGCGGG CAAAGTATGACCGATTATGCGATGAGC	CTTGCAGAAGCTGTGGTGGTGCAAATAATGG TACCACG
S7	*NmNEPS1-154SAGA*	ACCACCTCTGCTGGTGCAAGCCGTGGCGGG CAAAGTATGACCGATTATGCGATGAGC	CTTGCACCAGCAGAGGTGGTGCAAATAATGG TACCACG

figure number	Gene description	FW primer	RV primer
S7	*NmNEPS1-154SPTA*	ACCACCTCTCCAACAGCAAGCCGTGGCGGG CAAAGTATGACCGATTATGCGATGAGC	CTTGCTGTTGGAGAGGTGGTGCAAATAATGG TACCACG
S8	*NmNEPS1-154SATA-S198M*	TGACCCCGCTGGCGCAGCGTATGGGCCTGG CAACACCGGATGATTTTCAT	ACGCTGCGCCAGCGGGGTCAGCACCACCATT GGCGTCACGCAGTTAA
S8	*NmNEPS1-154SATA-S198L*	GCTGGCGCAGCGTATGG	CCATACGCTGCGCCAGCGGGGTCAGCACCAC TAATGGCGTCACGCAGTTAA
S8	*NmNEPS1-154SATA-S198P*	GCTGGCGCAGCGTATGG	CCATACGCTGCGCCAGCGGGGTCAGCACCAC AGGTGGCGTCACGCAGTTAA
S8	*NmNEPS1-154SATA-S198V*	GCTGGCGCAGCGTATGG	CCATACGCTGCGCCAGCGGGGTCAGCACCAC AACTGGCGTCACGCAGTTAA
S8	*NmNEPS1-154SATA-S198G*	GCTGGCGCAGCGTATGG	CCATACGCTGCGCCAGCGGGGTCAGCACCAC TCCTGGCGTCACGCAGTTAA
S8	*NmNEPS1-154SATA-S198A*	GCTGGCGCAGCGTATGG	CCATACGCTGCGCCAGCGGGGTCAGCACCAC TGCTGGCGTCACGCAGTTAA
S8	*NmNEPS1-154SATA-S198T*	GCTGGCGCAGCGTATGG	CCATACGCTGCGCCAGCGGGGTCAGCACCAC TGTTGGCGTCACGCAGTTAA
S8	*NmNEPS1-154SATA-S198C*	GCTGGCGCAGCGTATGG	CCATACGCTGCGCCAGCGGGGTCAGCACCAC ACATGGCGTCACGCAGTTAA
S9	*NsNEPS1B-151GAMS*	ACGACCGGTGCGATGTCGTCCAGGGGCGGG CAAAGCATGACGGACTATGTGATGTCGA	GGACGACATCGCACCGGTCGTGCAGATAATA GTGCCTCTC
S9	*NsNEPS1B-151SATS*	ACGACCAGCGCGACATCGTCCAGGGGCGGG CAAAGCATGACGGACTATGTGATGTCGA	GGACGATGTCGCGCTGGTCGTGCAGATAATA GTGCCTCTC
S9	*NsNEPS1B-151SATA*	ACGACCAGCGCGACAGCTTCCAGGGGCGGG CAAAGCATGACGGACTATGTGATGTCGA	GGAAGCTGTCGCGCTGGTCGTGCAGATAATA GTGCCTCTC
S9	*NsNEPS1B-S195L*	TCACGCCGCTCGCCCAAAGGATGGGGTTTTC CACGCCCGATGATTTCCATACTCATTTTG	CCTTTGGGCGAGCGGCGTGATCACCAATAAC GGCGACACGCAGTTAACCCTAA
S9	*NsNEPS1B-151GSSA*	ACGACCGGTTCTTCAGCTTCCAGGGGCGGGC AAAGCATGACGGACTATGTGATGTCGA	GGAAGCTGAAGAACCGGTCGTGCAGATAATA GTGCCTCTC
S9	*NsNEPS1B-151AAMS*	ACGACCGCTGCGATGTCGTCCAGGGGCGGG CAAAGCATGACGGACTATGTGATGTCGA	GGACGACATCGCAGCGGTCGTGCAGATAATA GTGCCTCTC
S9	*NsNEPS1B-151ASTA*	ACGACCGCGTCTACAGCATCCAGGGGCGGG CAAAGCATGACGGACTATGTGATGTCGA	GGATGCTGTAGACGCGGTCGTGCAGATAATA GTGCCTCTC
S9	*NsNEPS1B-151ASMA*	ACGACCGCAAGCATGGCTTCCAGGGGCGGG CAAAGCATGACGGACTATGTGATGTCGA	GGAAGCCATGCTTGCGGTCGTGCAGATAATA GTGCCTCTC

NEPS	NcL1	NmL1	NsL	Nc3A	Nc3B	Nm3	Ns2	Nm2	Nc2	Nm5	Nc5A	Ns4A	Ns4B	Nm4	Nc4	Nc1	Nm1	Ns1A	Ns1B
MpIDPH	73	70	70	66	65	66	72	70	69	64	64	67	66	63	65	63	62	66	65
NcNEPSL1	100	89	78	69	68	69	71	69	68	67	67	70	68	65	66	68	67	72	70
NmNEPSL1		100	77	68	68	68	69	67	66	65	65	68	66	64	64	66	65	69	67
NsNEPSL			100	66	66	67	68	67	66	63	63	67	65	64	63	66	64	68	66
NcNEPS3A				100	92	93	72	69	68	65	64	68	66	64	65	64	64	68	67
NcNEPS3B					100	95	72	68	67	64	62	66	65	63	65	64	64	67	66
NmNEPS3						100	72	69	68	65	63	68	66	64	66	65	65	68	68
NsNEPS2							100	90	89	72	71	75	73	70	72	72	71	75	74
NmNEPS2								100	95	70	68	73	71	69	72	72	71	73	73
NcNEPS2									100	70	68	72	71	68	71	71	71	72	71
NmNEPS5										100	95	80	80	77	79	81	81	80	79
NcNEPS5A											100	78	79	75	77	79	79	80	79
NsNEPS4A												100	96	88	89	87	83	88	86
NsNEPS4B													100	88	89	85	81	84	83
NmNEPS4														100	92	83	80	81	80
NcNEPS4															100	85	82	84	82
NcNEPS1																100	93	89	90
NmNEPS1																	100	86	87
NsNEPS1A																		100	94
NsNEPS1B																			100

Supplementary Figure 1: Pairwise comparison of amino acid identities of NEPS sequences. Abbreviated sequences at the top consist of the same names of each enzyme in the left column excluding the abbreviation "NEPS". Lighter shades of red correspond to lower protein sequence identity.

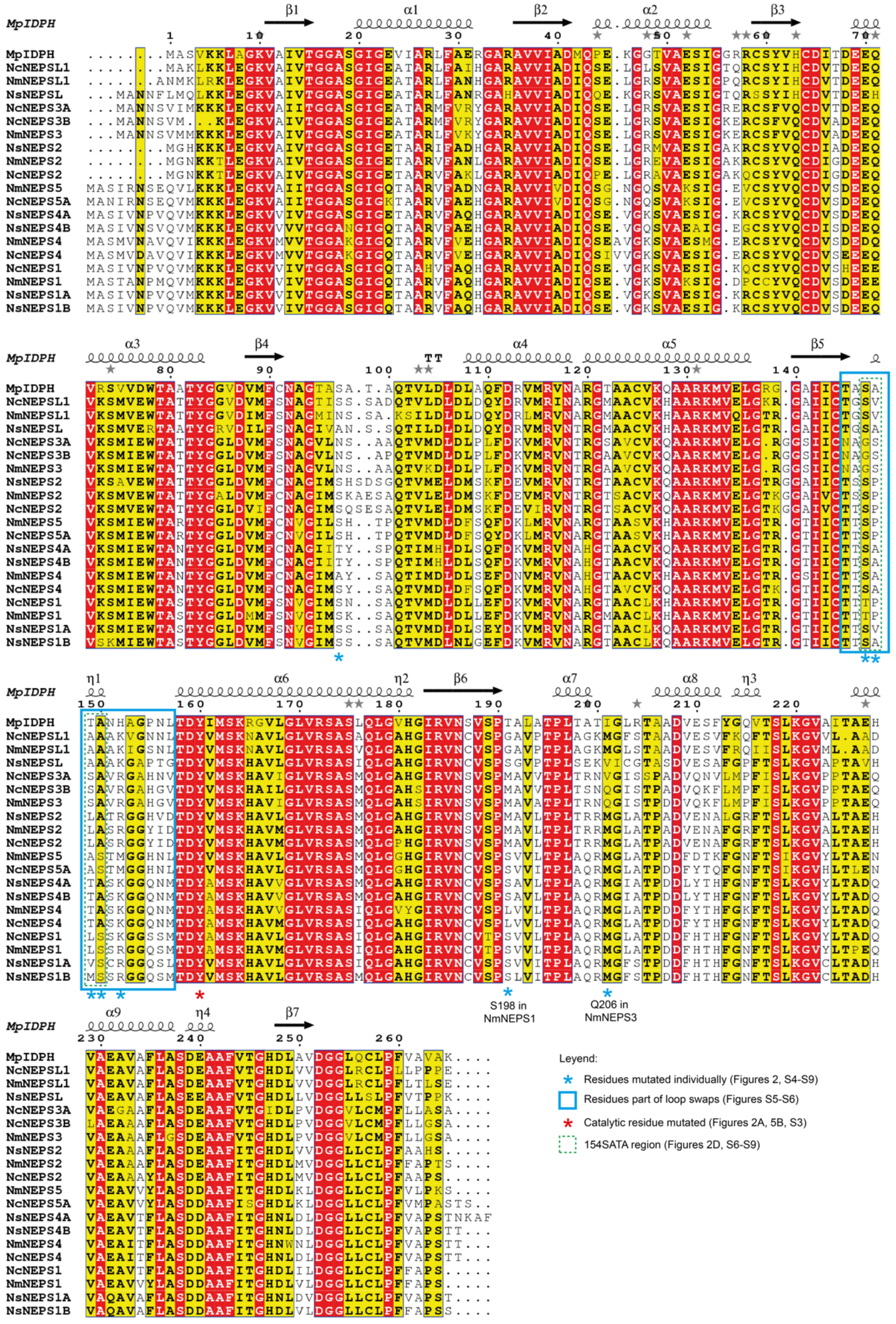

Supplementary Figure 2: Sequence alignment of all NEPS enzymes from three species: *Nepeta sibirica* (Ns), *Nepeta cataria* (Nc), and *Nepeta mussinii* (Nm). MpIDPH is a relative enzyme from *Mentha piperita* (spearmint). Positions with identical residues are highlighted in red, whereas similar residues are highlighted in yellow. Additional categories related to this study are described in the legend.

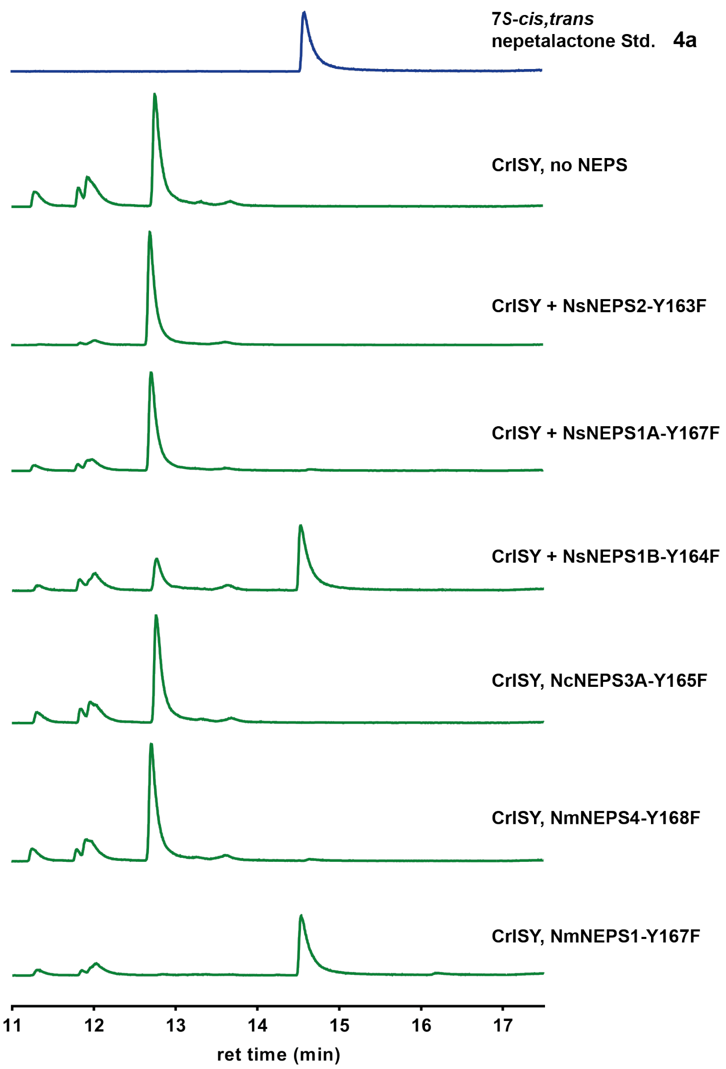

Supplementary Figure 3: NEPS catalytic tyrosine mutations to phenylalanine leads to various degrees of disruption of oxidation. NsNEPS2 is shown completely abolishing oxidation to 7*S-cis-trans* nepetalactone **4a** while maintaining cyclization activity (as can be seen by the disappearance of iridodials). NsNEPS1A has similarly lost oxidation activity and maintained cyclization. On the other hand, NsNEPS1B and NmNEPS1 still are able to oxidize to 7*S-cis-trans* nepetalactone **4a**, while NcNEPS3A and NmNEPS4 appear inactive for both cyclization and oxidation. Results were repeated twice independently with similar results.

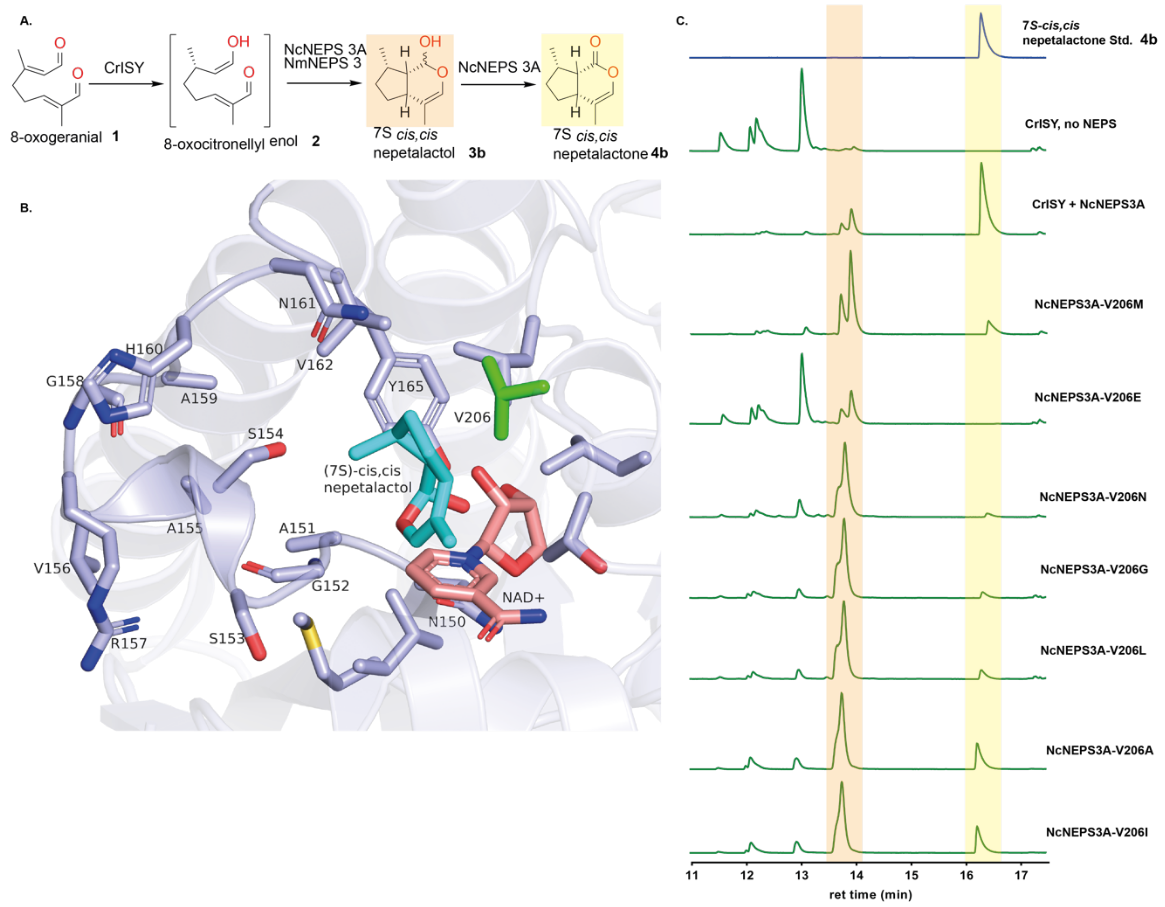

Supplementary Figure 4: Mutagenesis of NcNEPS3A residue V206. A. NcNEPS3A and NmNEPS3 native activities. B. Crystal structure model showing V206 residue (highlighted in green, annotated as V206) and its location relative to NAD+ and a manually docked 7*S*-*cis*-*cis* nepetalactol **3b** molecule. C. Oxidation activity to **4b** can be changed with various V206 point mutations. Highlighted parts of chromatograms represent the molecular structure highlighted with the same color. Results were repeated twice independently with similar results.

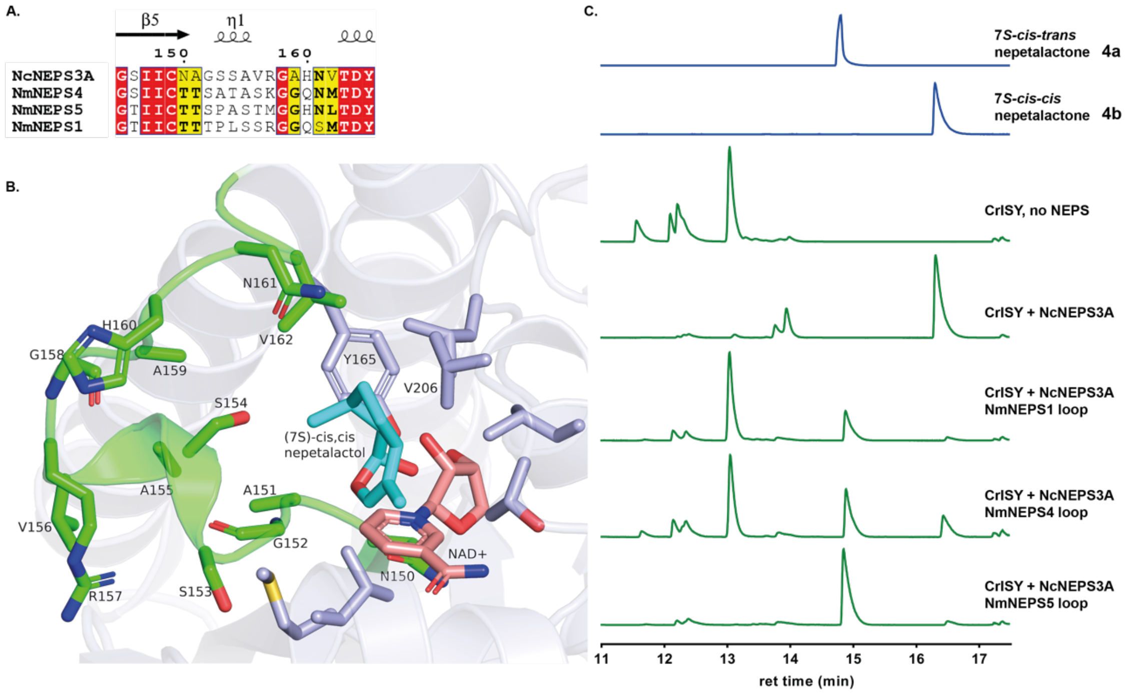

Supplementary Figure 5: Loop 150-162 swap variants generated in NcNEPS3A. A. Amino acid sequence alignment showing the 150-162 loop region to be swapped. B. Crystal structure model showing the 150-162 loop (highlighted in green, annotated as A151, G152, S153, S154, V156, R157, G158, A159, H160, N161, V162) and its location relative to NAD+ and a manually docked 7*S*-*cis*-*cis* nepetalactol **3b** molecule. C. Cyclization and oxidation activities in NcNEPS3A change when the 150-162 loop is replaced with those from NmNEPS1 and NmNEPS4. Results were repeated twice independently with similar results.

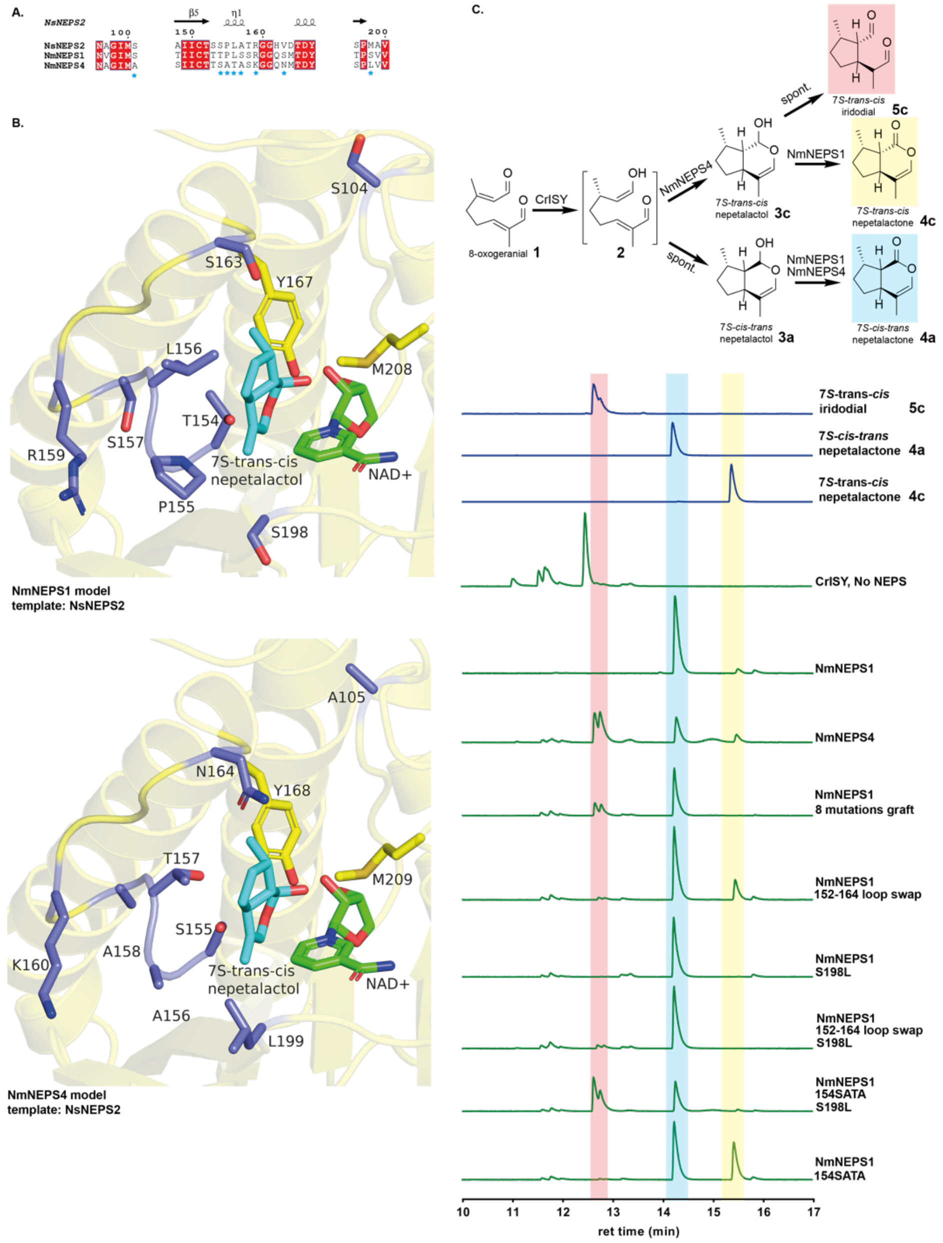

Supplementary Figure 6: Engineering *7S-trans-cis* **3c** cyclization in NmNEPS1. A. Partial sequence alignment of the active site regions with highlighted residue differences (blue asterisks). B. Crystal structure models of NmNEPS1 and NmNEPS4 based on NsNEPS2 with 7*S-trans-cis* nepetalactol **3c** manually docked into the active site (light blue), NAD+ highlighted (green, annotated as NAD+) and residues that are different between the two enzymes (violet). C. Enzyme roles in cyclization (NmNEPS4) and oxidation (NmNEPS1) of 7*S-trans-cis* nepetalactol **3c** and enzymatic assays of NmNEPS1 variants coupled with 8-oxogeranial **1** and CrISY. Complete graft of all 8 active site residue differences from NmNEPS4 into NmNEPS1 shows some 7*S-trans-cis* nepetalactol **3c** cyclization gained (which spontaneously opens into 7*S-trans-cis* iridodial **5c**) but oxidation activity was lost. Loop swap of the 152-164 region shows some gained activity for 7*S-trans-cis* nepetalactone **4c** but remains a minor product. Adding S198L mutation to the loop swap disrupts cyclization of 7*S-trans-cis* nepetalactol **3c** gained. When only the 154SATA residues are grafted, 7*S-trans-cis* nepetalactol **3c** production is improved and its subsequent oxidation to 7*S-trans-cis* nepetalactone **4c** is maintained. Highlighted parts of chromatograms

represent the molecular structure highlighted with the same color. Results were repeated twice independently with similar results.

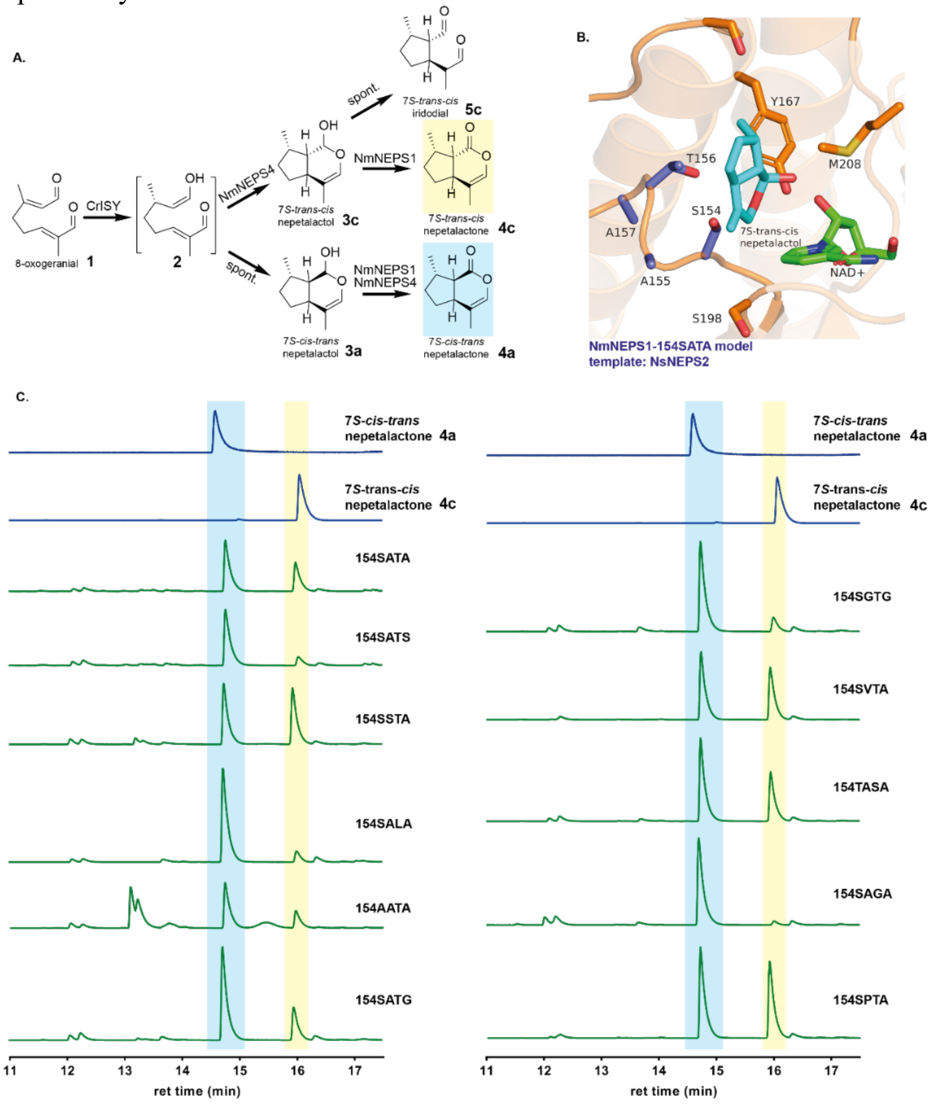

Supplementary Figure 7: 154SATA region variants generated in NmNEPS1. A. Enzyme roles in cyclization (NmNEPS4) and oxidation (NmNEPS1) of 7*S*-*trans*-*cis* nepetalactol **3c**. B. Crystal structure model of NmNEPS1 154SATA variant based on NsNEPS2 with 7*S*-*trans*-*cis* nepetalactol **3c** docked into the active site (light blue), NAD+ highlighted (green, annotated as NAD+) and 154SATA residues highlighted (violet). C. Variations in the 154SATA loop region have direct impact in cyclization and oxidation of 7*S*-*trans*-*cis* nepetalactol **3c**. S154A mutation does not appear to disrupt cyclization but oxidation to 7*S*-*trans*-*cis* nepetalactone **4c** was disrupted. S156T on the other hand, did not change the profile suggesting that a polar group in 156 position is needed for stabilization of 7*S*-*trans*-*cis* nepetalactol **3c** for oxidation. Position 155 changes made did not disrupt cyclization or oxidation but A155S, A155V and A155P had positive impact towards production of 7*S*-*trans*-*cis* nepetalactone **4c**. Changing position 156 to non-polar Gly and Leu had a large impact on 7*S*-*trans*-*cis* nepetalactol **3c** cyclization, suggesting that this residue is involved in cyclization activity. Finally, although position 157 does not appear to be directly in contact with the substrate (part B) it seems to have an impact on the overall loop stability, given that mutations A157G and A157S do have detrimental impact on 7*S*-*trans*-*cis* nepetalactone **4c** production. Highlighted parts of chromatograms represent the molecular structure highlighted with the same color. Results discussed in the text (such as 154SATA, and 154SVTA mutants) were repeated at least twice times independently with similar results.

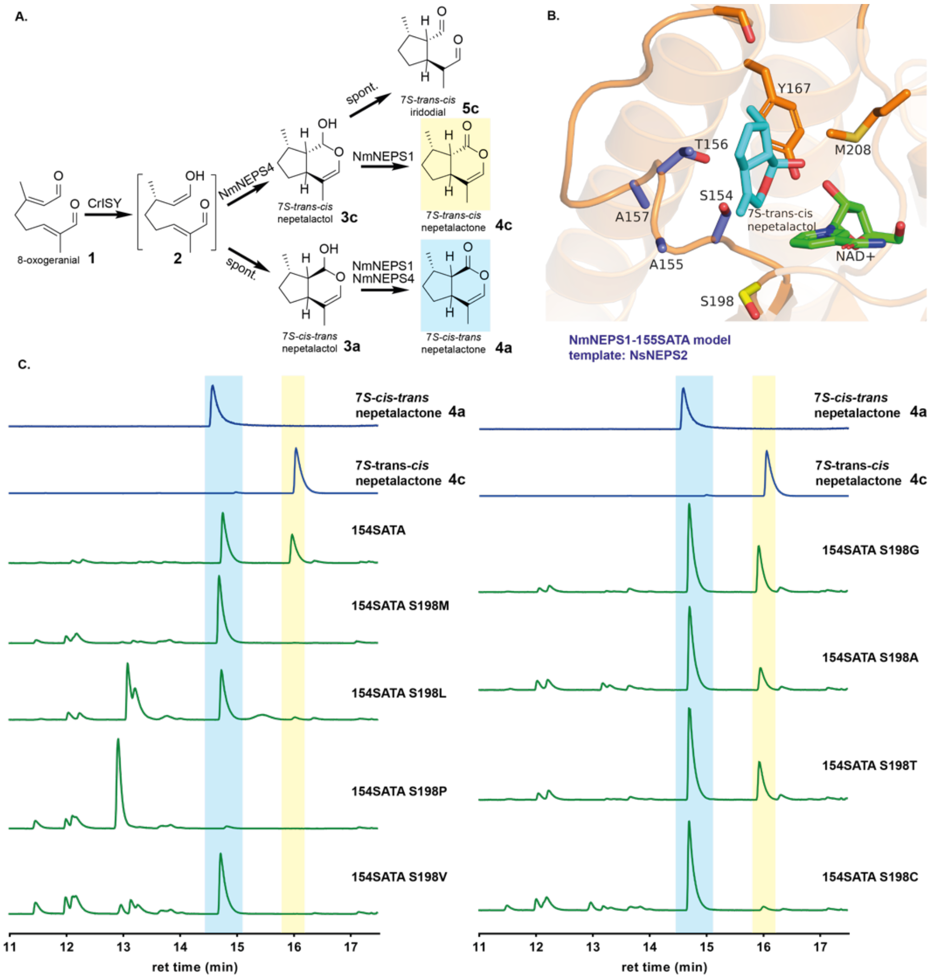

Supplementary Figure 8: Variations of the residue S198 in the NmNEPS1-154SATA variant enzyme. A. Enzyme roles in cyclization (NmNEPS4) and oxidation (NmNEPS1) of 7*S*-*trans-cis* nepetalactol **3c**. B. Crystal structure model of NmNEPS1 154SATA variant based on NsNEPS2 with 7S-*trans-cis* nepetalactol **3c** docked into the active site (light blue), NAD+ highlighted (green, annotated as NAD+) and S198 residue highlighted (yellow). C. Variations of S198 residue of NmNEPS1-154SATA variant have an impact in 7*S*-*trans-cis* nepetalactone **4c** production. Highlighted parts of chromatograms represent the molecular structure highlighted with the same color. Results discussed in the text (such as S198L mutant) were repeated three times independently with similar results.

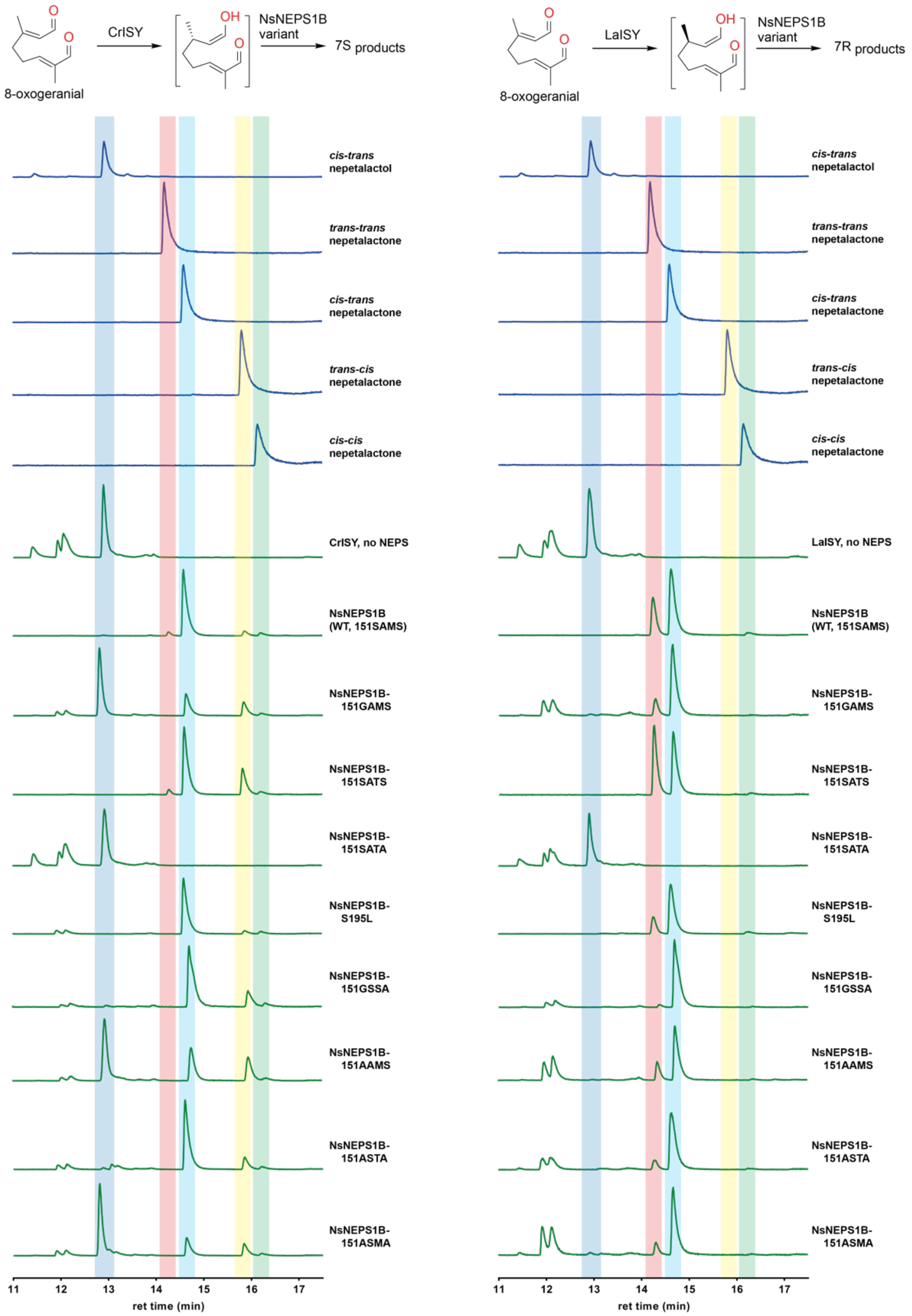

Supplementary Figure 9: NsNEPS1B variants tested with stereo-divergent iridoid synthases (ISY). Achiral GC-MS traces showing the impact of various mutations in NsNEPS1B on the product profile for 7*S* (left) and 7*R* (right) isomers. Highlighted parts of chromatograms represent the standard peaks highlighted with the same color. This was an initial screen to engineer *trans,trans* activity. Since all results were negative, the assays were only performed once and were not investigated further.

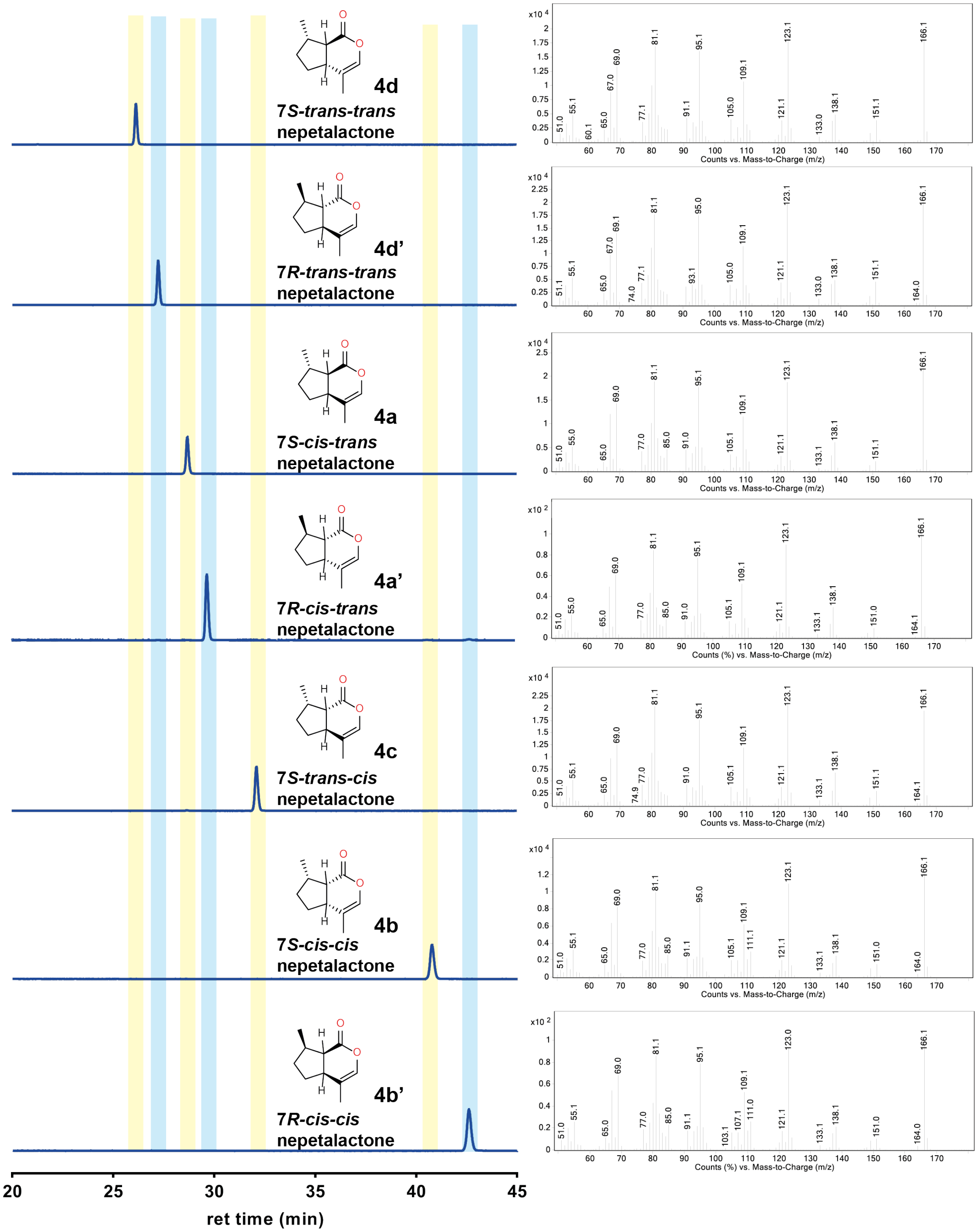

Supplementary Figure 10: Chiral column chromatograms and mass spectra of nepetalactone standards. These are standards and have been measured more than three times over many months.

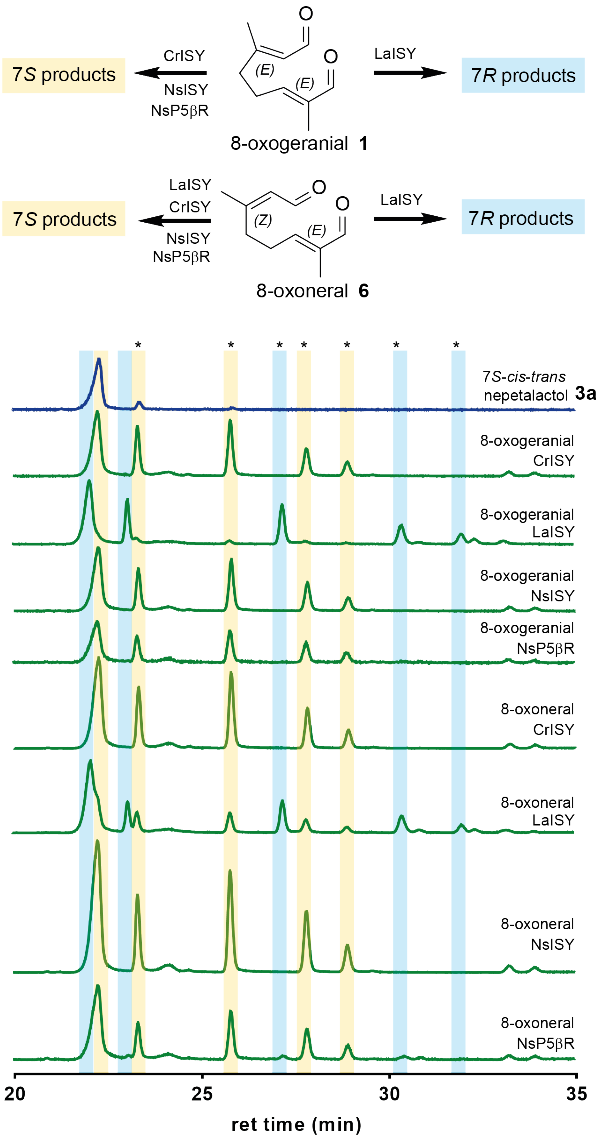

Supplementary Figure 11: Influence of 8-oxoneral as a substrate to ISY in the resulting product profile. 8-oxogeranial and 8-oxoneral were assayed side by side with *N. sibirica* ISY and P5ßR, as well as CrISY and LaISY in order to see the impact of the substrate stereochemistry in the product profile. For CrISY, NsISY and NsP5ßR, both substrates result in 7*S* *cis-trans* nepetalactol **3a** and iridodials (asterisks), indicating no change in profile. In the case of LaISY, while 8-oxogeranial results in 7*R* products, 8-oxoneral leads to both 7*S* and 7*R* products. Highlighted parts of chromatograms represent 7*S* products (light yellow) and 7*R* products (light blue). LaISY and CrISY were each performed twice with independent results.

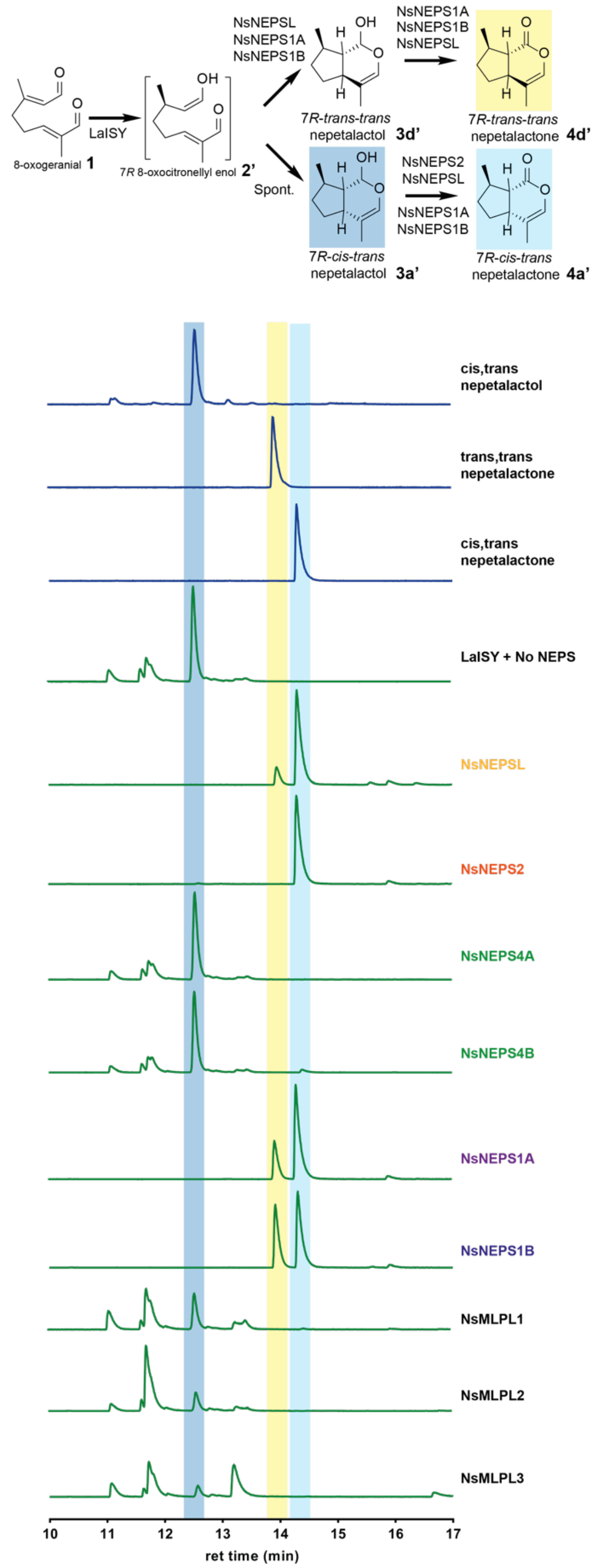

Supplementary Figure 12: Achiral GC-MS data of *N. sibirica* NEPS and MLPL assayed in combination with 8-oxogeranial **1** and 7*R*-specific iridoid synthase from *Lamium album*, LaISY. Highlighted parts of chromatograms represent the molecular structure highlighted with the same color. Results were repeated twice independently with similar results.

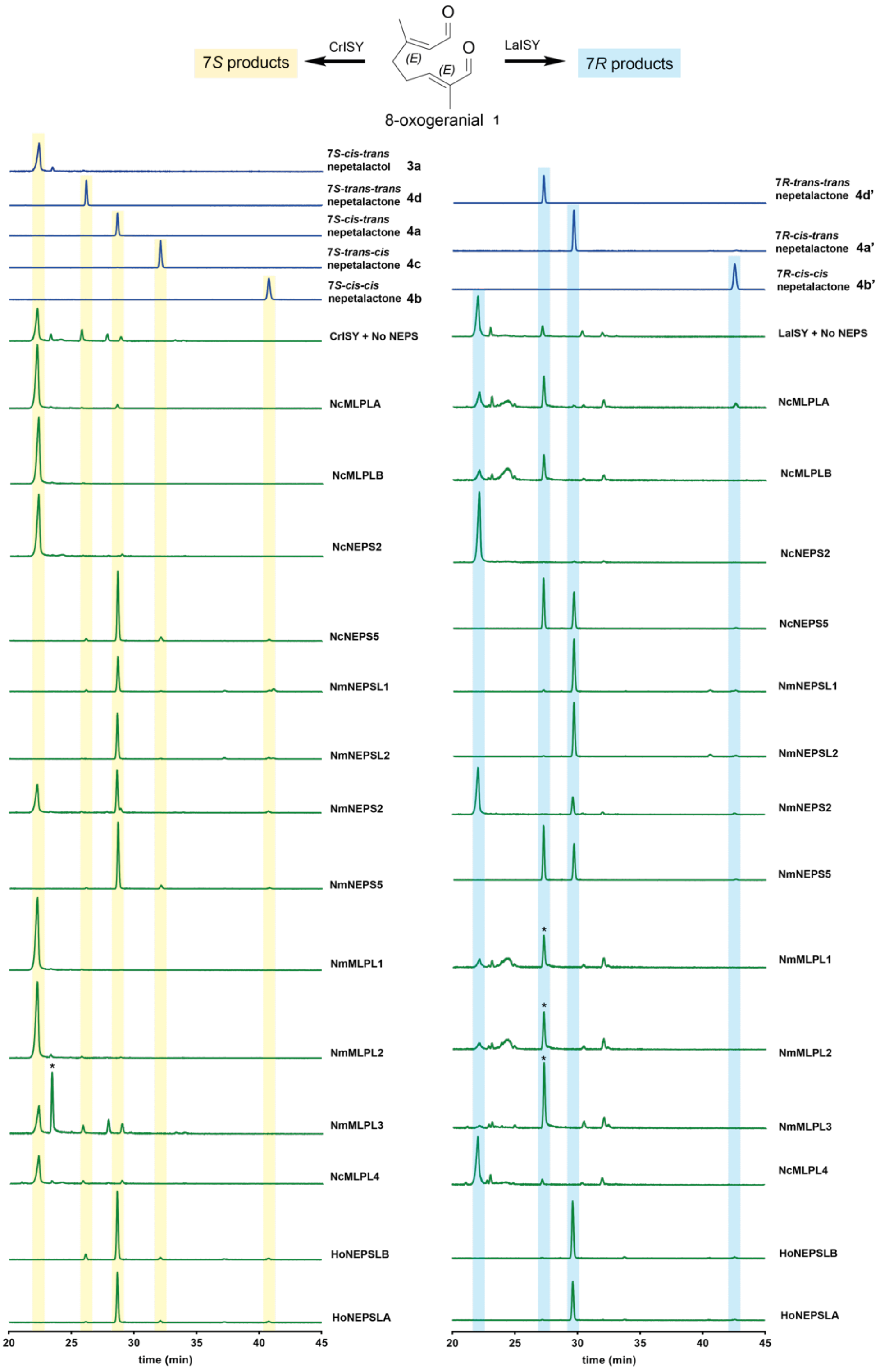

Supplementary Figure 13: Chiral GC-MS survey of *N. mussinii, N. cataria* and *H. officinalis* selected NEPS and MLPL assayed in combination with 8-oxogeranial and both, 7*S*-specific iridoid synthase CrISY and 7*R*-specific iridoid synthase LaISY. Highlighted parts of chromatograms represent 7*S* products (light yellow) and 7*R* products (light blue). Asterisks indicate iridodials. Results were repeated twice independently with similar results.

Supplementary method:
Nepetalactone epimerization and purification
The base 1,8-diaza-bicyclo[5.4.0]undec-7-ene (DBU) was added (126 mg, 0.846 mmol) to a stirred solution of 7*S*-*cis*-*cis* nepetalactone (126 mg, 0.758 mmol) in toluene (6 mL) at room temperature and the resulting mixture was refluxed for 24h. After cooling to room temperature, the reaction was purified by silica gel column chromatography (PE/ethyl acetate = 20/1) to afford 7*S*-*trans*-*trans* **4d** nepetalactone (9.5 mg, 8%) and recover 7*S*-*cis*-*cis* nepetalactone **4b** (100 mg).

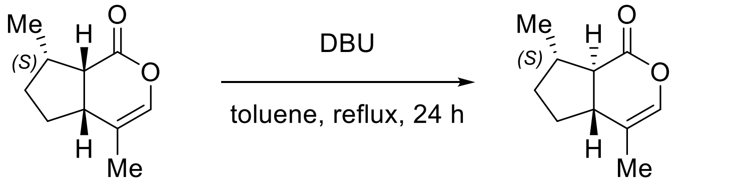

7*S*-*cis*-*cis* **4b** 7*S*-*trans*-*trans* **4d**

DBU (8.9 uL mg, 0.0596 mmol) was added to a stirred solution of 7*R*-*trans*-*trans* nepetalactone **4d'** (9 mg, 0.0542 mmol) in toluene (1 mL) at room temperature and the resulting mixture was refluxed for 12h. After cooling to room temperature, the reaction was purified by silica gel column chromatography (PE/ethyl acetate = 20/1) to afford 7*R*-*cis*-*cis* nepetalactone **4b'** (4.5 mg, 47%).

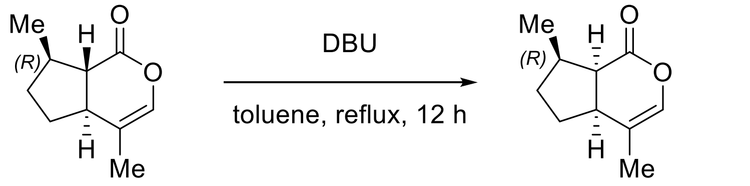

7*R*-*trans*-*trans* **4d'** 7*R*-*cis*-*cis* **4b'**

NMR
NMR spectra were measured on a 400 MHz Bruker Avance III HD (Bruker Biospin GmbH, Rheinstetten, Germany) (Supplementary Figures 13-17). $CDCl_3$ was used as solvent. NMR spectra were referenced to the residual solvent signals at δ_H 7.26 ppm and δ_C 77.0 ppm. For spectrometer control and data processing Bruker TopSpin ver. 3.6.1 was used. Mass spectral data for these compounds are also provided in Supplementary Figure 10.

Data of 7*S*-*trans*-*trans* nepetalactone **4d**: ^{1}H NMR (400 MHz, $CDCl_3$) δ 6.25 (dq, *J* = 3.1, 1.6 Hz, 1H), 2.58-2.48(m, 1H), 2.30-2.17 (m, 1H), 2.12-2.01 (m, 1H), 1.98-1.83 (m, 2H), 1.69 (t, *J* = 1.5 Hz, 3H), 1.51-1.36 (m, 2H), 1.19 (d, *J* = 6.6 Hz, 3H);
^{13}C NMR (100 MHz, $CDCl_3$) δ171.5, 136.3, 120.6, 52.4, 41.8, 32.5, 31.6, 25.6, 20.4, 14.0.

Data of 7*R*-*cis*-*cis* nepetalactone **4b'**: ^{1}H NMR (400 MHz, $CDCl_3$) δ 6.19-6.16 (m, 1H), 3.10 (t, *J* = 9.6 Hz, 1H), 2.85 -2.75 (m, 1H), 2.68-2.56 (m, 1H), 1.96-1.74 (m, 3H), 1.60 (t, *J* = 1.2 Hz, 3H), 1.39-1.28 (m, 1H), 1.00 (d, *J* = 7.2 Hz, 3H);
^{13}C NMR (100 MHz, $CDCl_3$) δ 170.2, 134.3, 115.5, 46.2, 39.4, 38.4, 32.7, 30.4, 17.2, 14.8.

Data of 7*S*-c*is*-c*is* nepetalactone **4b**: ^{1}H NMR (400 MHz, $CDCl_3$) δ 6.21–6.14 (m, 1H), 3.10 (t, *J* = 9.6 Hz, 1H), 2.85–2.75 (m, 1H), 2.67–2.56 (m, 1H), 1.93–1.76 (m, 3H), 1.60 (t, *J* = 1.3 Hz, 3H), 1.37-1.31 (m, 1H), 1.00 (d, *J* = 7.2 Hz, 3H);
^{13}C NMR (100 MHz, $CDCl_3$) δ 170.2, 134.2, 115.5, 46.2, 39.4, 38.4, 32.7, 30.4, 17.2, 14.8.

Data of 7*R*-*trans*-*trans* nepetalactone **4d'**: ^{1}H NMR (400 MHz, $CDCl_3$) δ 6.25 (dq, *J* = 3.2, 1.6 Hz, 1H), 2.59–2.47 (m, 1H), 2.28-2.19 (m, 1H), 2.12–2.01 (m, 1H), 1.98–1.84 (m, 2H), 1.69 (t, *J* = 1.6 Hz, 3H), 1.51–1.36 (m, 2H), 1.19 (d, *J* = 6.6 Hz, 3H);
^{13}C NMR (100 MHz, $CDCl_3$) δ 171.5, 136.3, 120.6, 52.5, 41.8, 32.5, 31.6, 25.7, 20.4, 14.0.

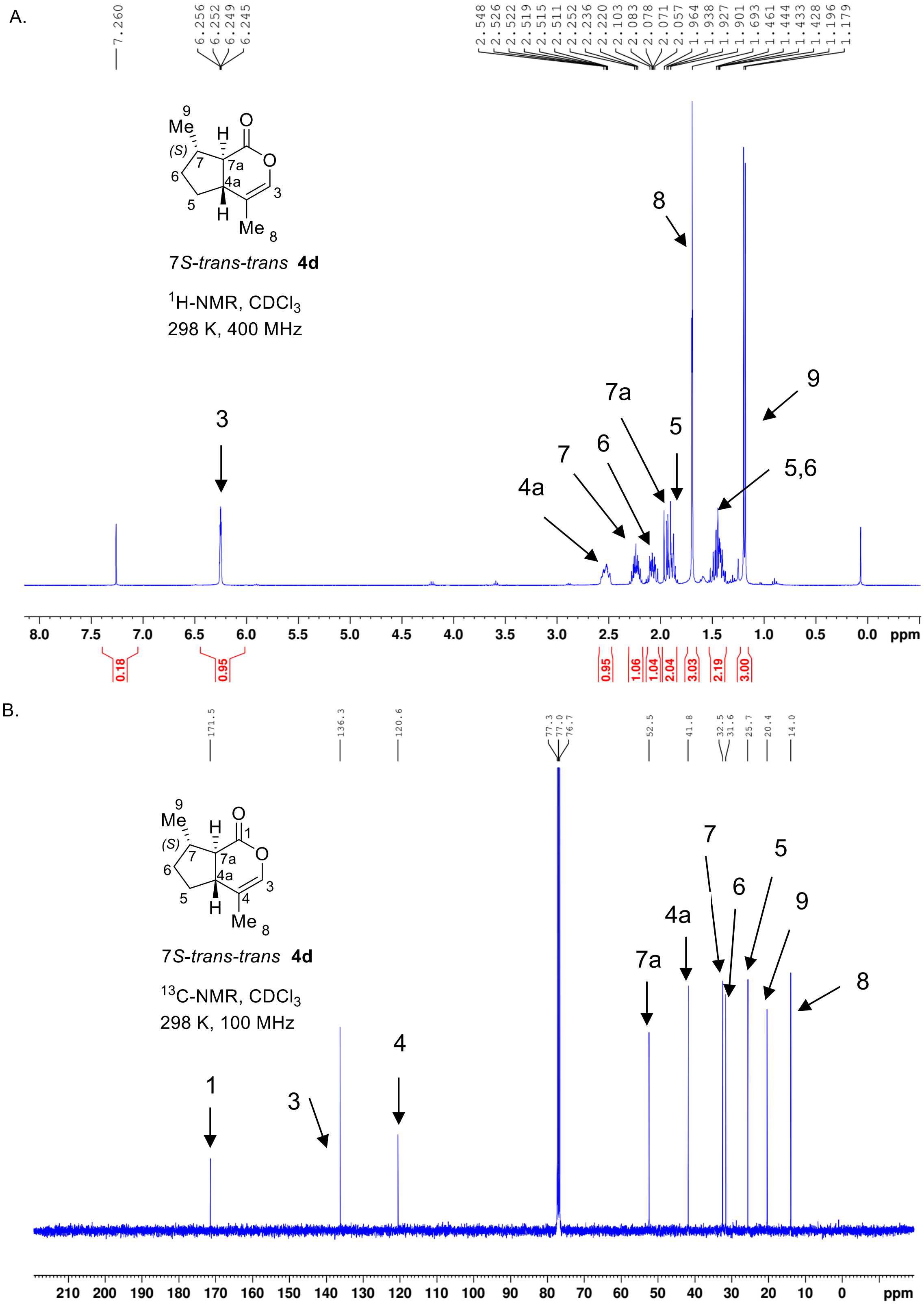

Supplementary Figure 14: Proton (A) and Carbon (B) NMR of 7*S*-*trans*-*trans* nepetalactone **4d**.

A.

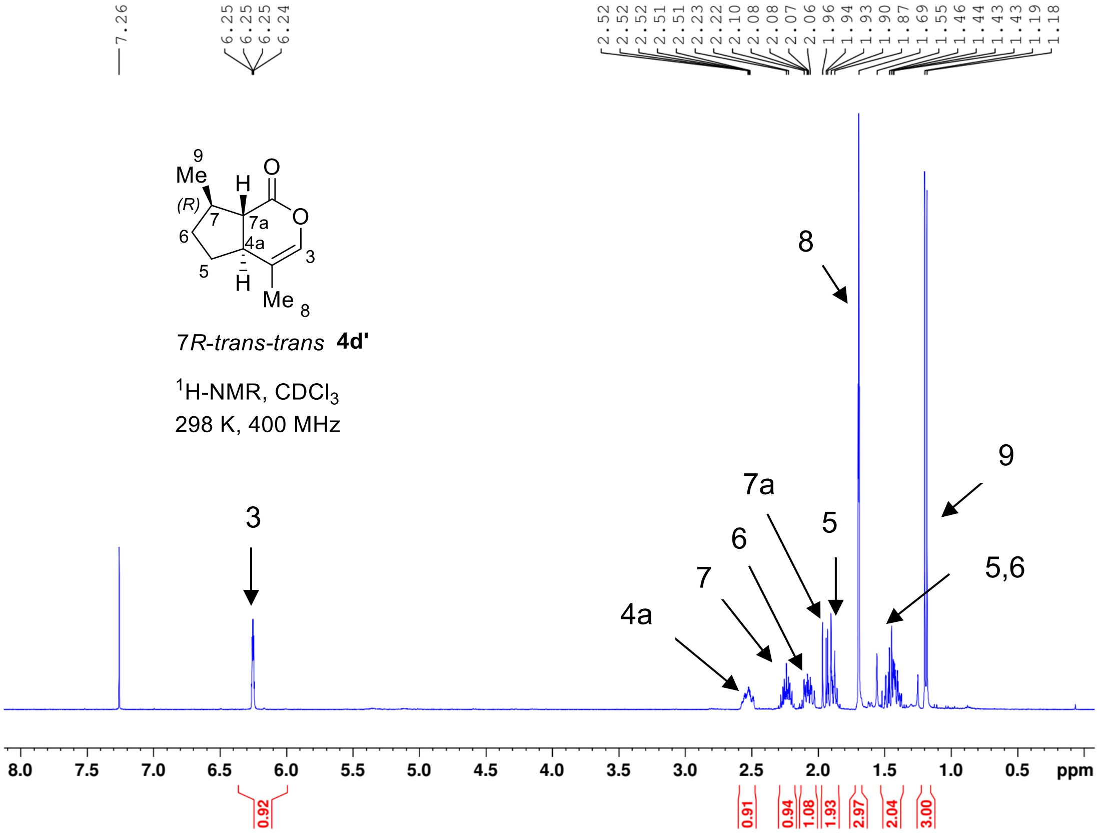

7R-trans-trans 4d'
^{1}H-NMR, $CDCl_3$
298 K, 400 MHz
ppm

B.

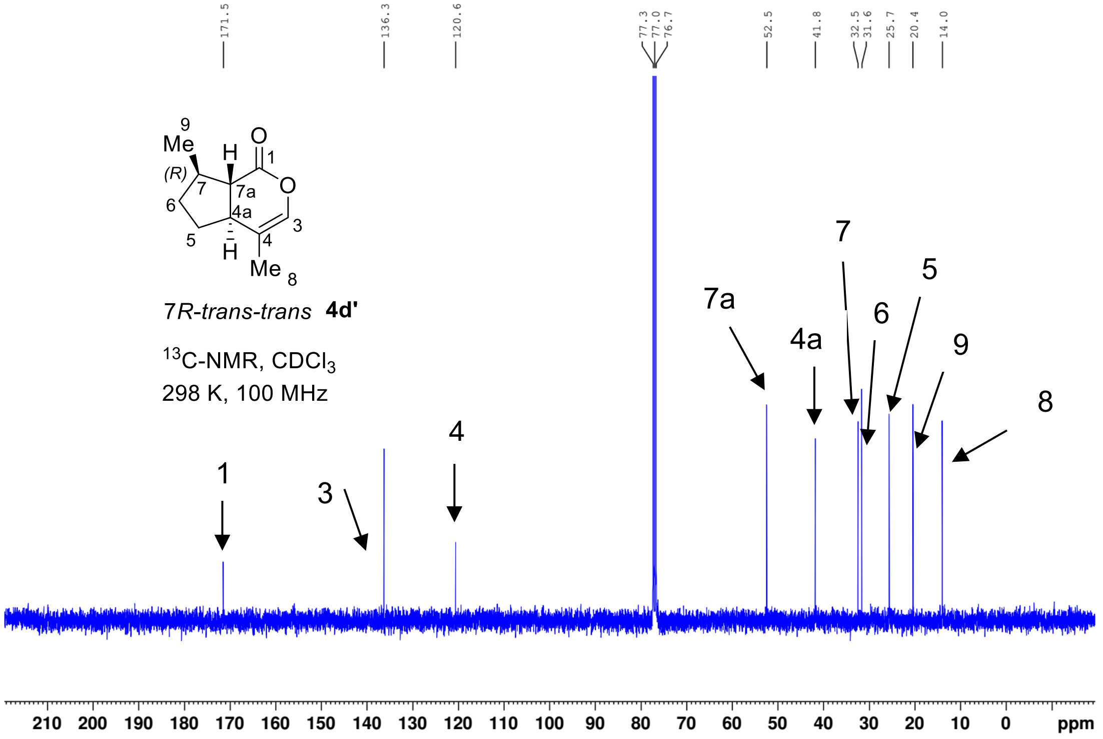

7R-trans-trans 4d'
^{13}C-NMR, $CDCl_3$
298 K, 100 MHz
ppm

Supplementary Figure 15: Proton (A) and Carbon (B) NMR of 7*R* *trans-trans* nepetalactone **4d'**.

A.

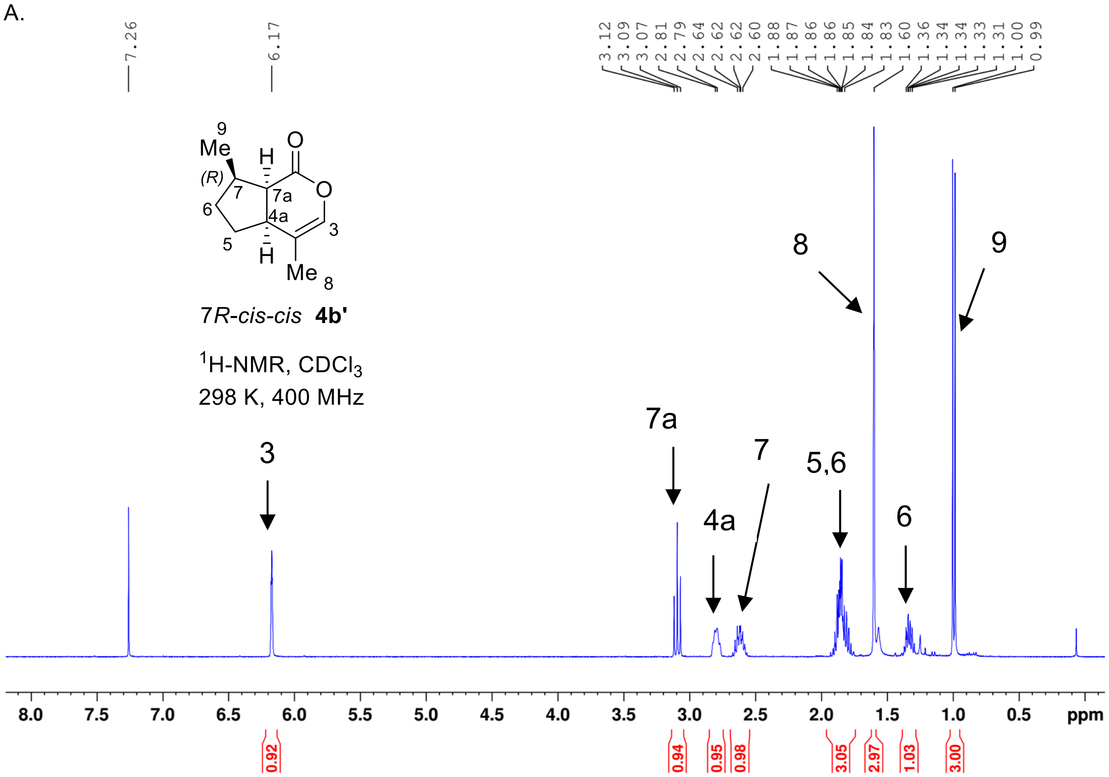

B.

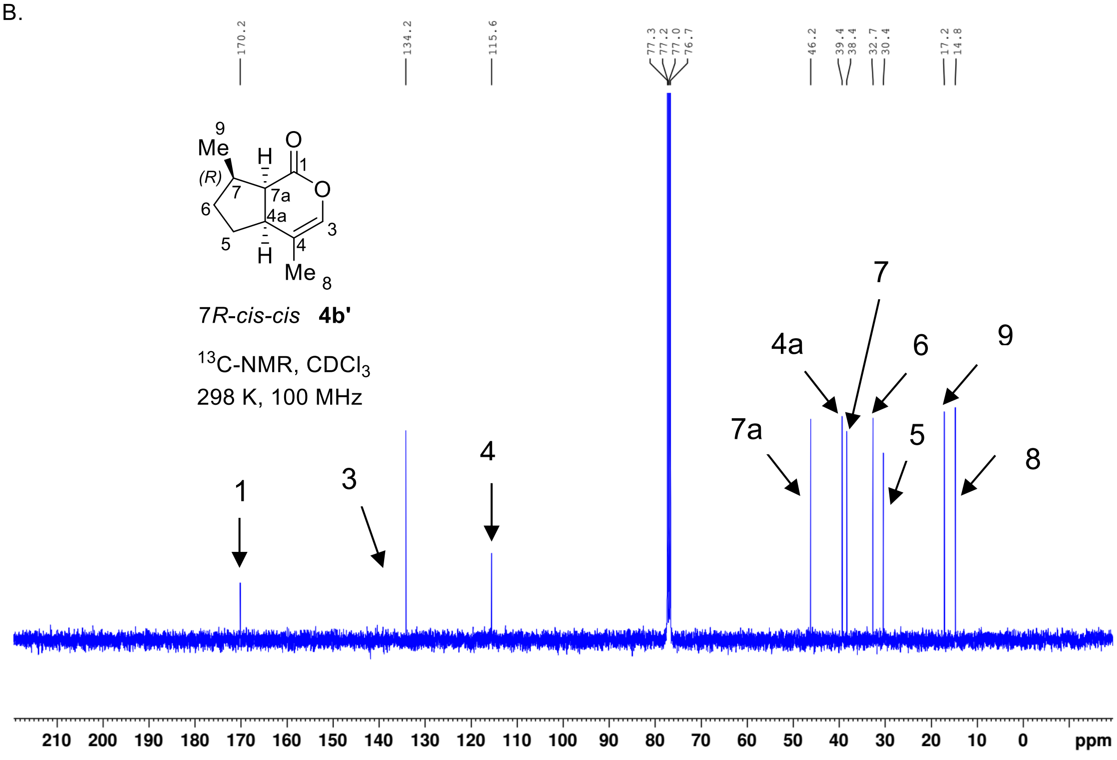

Supplementary Figure 16: Proton (A) and Carbon (B) NMR of 7*R* *cis-cis* nepetalactone **4b'**.

A.

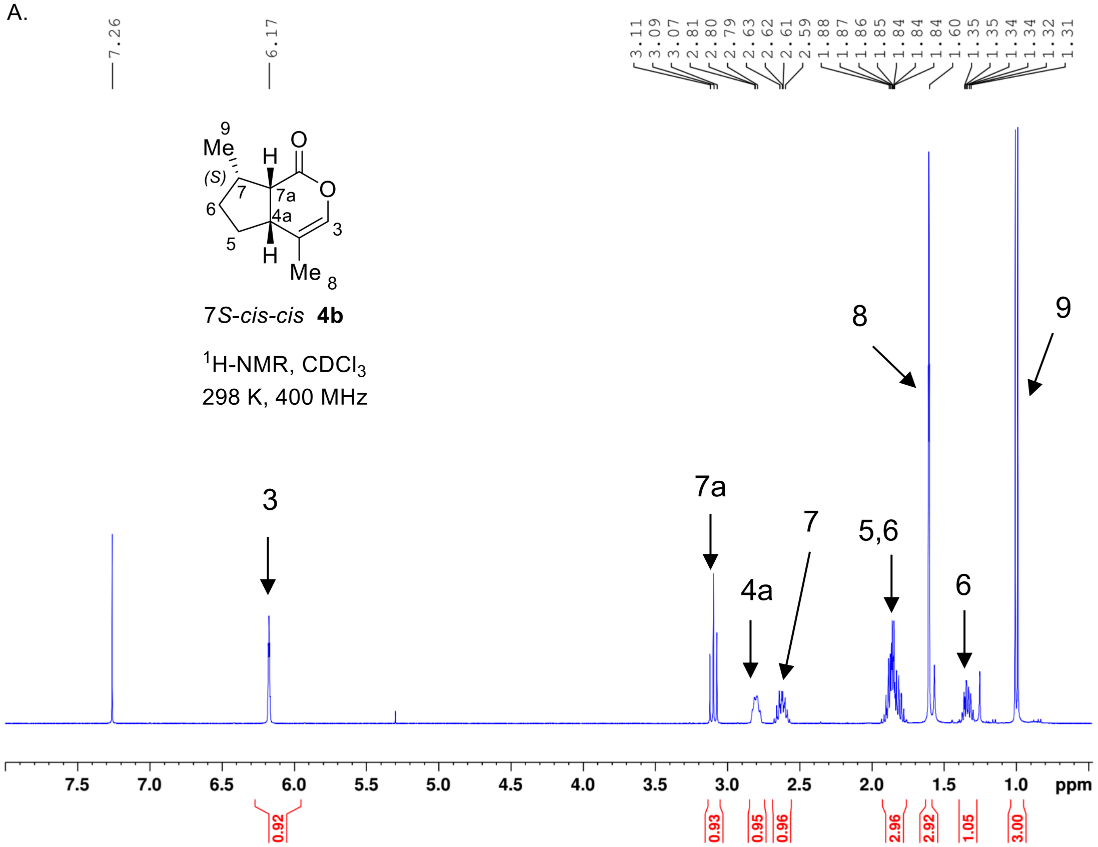

B.

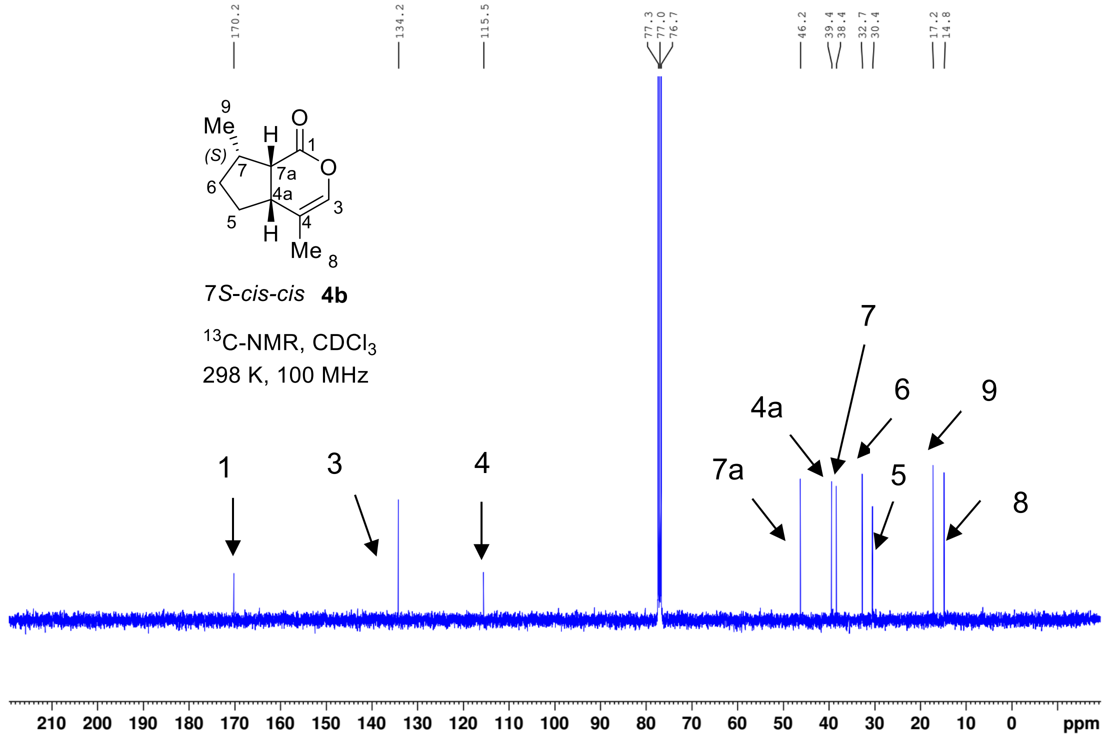

Supplementary Figure 17: Proton (A) and Carbon (B) NMR of 7*S*-*cis*-*cis* nepetalactone **4b**.

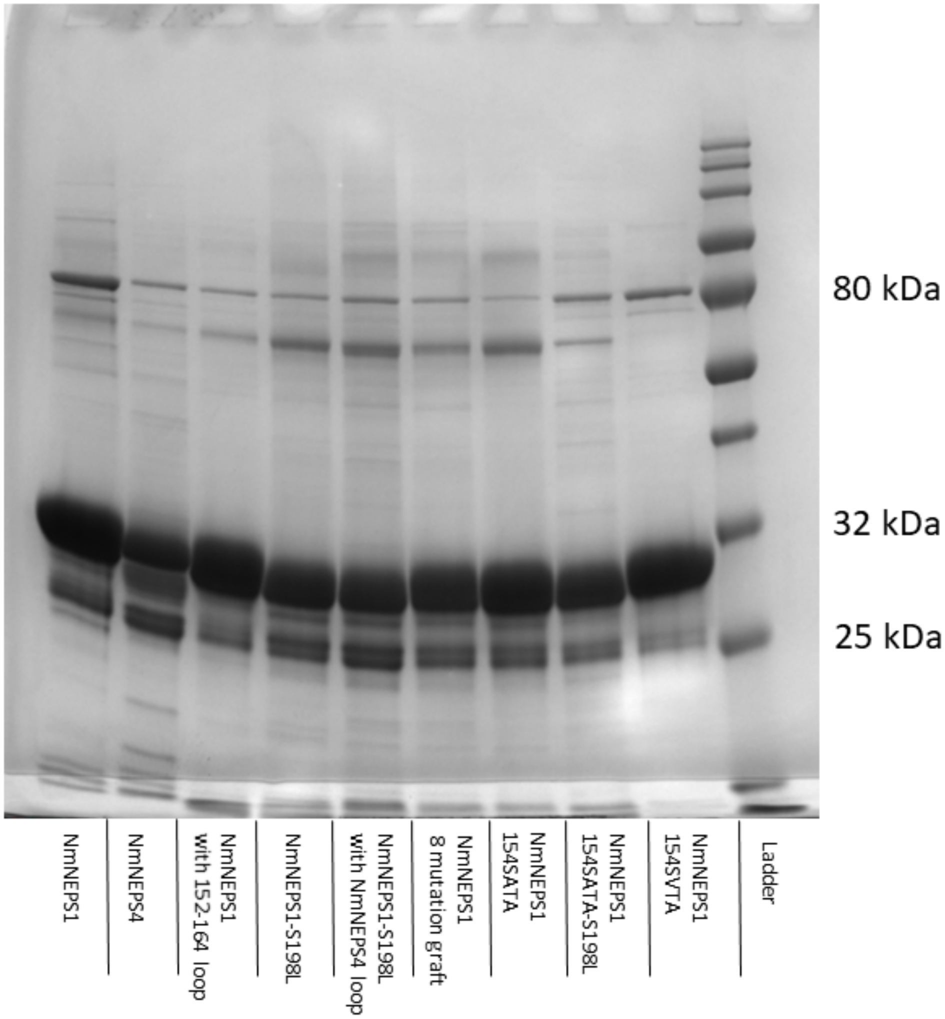

Supplementary Figure 18: Representative SDS-PAGE gel of proteins purified for Figures 2D and S6. The major band at 32 kDa represents the protein of interest. These proteins were expressed in E. coli and analyzed by SDS-PAGE at least twice.
