# Supplementary material for: Biocatalytic routes to stereo-divergent iridoids
Source: Nat Commun. 2022 Aug 11;13:4718. doi: 10.1038/s41467-022-32414-w (PMC9372074; doi:10.1038/s41467-022-32414-w)
Supplement: Supplementary file 3 — Reporting Summary [file 41467_2022_32414_MOESM3_ESM.pdf]

nature portfolio

Corresponding author(s): Sarah E. O'Connor

Last updated by author(s): 2022/07/22

Reporting Summary

Nature Portfolio wishes to improve the reproducibility of the work that we publish. This form provides structure for consistency and transparency in reporting. For further information on Nature Portfolio policies, see our Editorial Policies and the Editorial Policy Checklist.

Statistics

For all statistical analyses, confirm that the following items are present in the figure legend, table legend, main text, or Methods section.

n/a	Confirmed	
[ ]	[x]	The exact sample size (*n*) for each experimental group/condition, given as a discrete number and unit of measurement
[x]	[ ]	A statement on whether measurements were taken from distinct samples or whether the same sample was measured repeatedly
[x]	[ ]	The statistical test(s) used AND whether they are one- or two-sided<br>*Only common tests should be described solely by name; describe more complex techniques in the Methods section.*
[x]	[ ]	A description of all covariates tested
[x]	[ ]	A description of any assumptions or corrections, such as tests of normality and adjustment for multiple comparisons
[ ]	[x]	A full description of the statistical parameters including central tendency (e.g. means) or other basic estimates (e.g. regression coefficient) AND variation (e.g. standard deviation) or associated estimates of uncertainty (e.g. confidence intervals)
[x]	[ ]	For null hypothesis testing, the test statistic (e.g. *F*, *t*, *r*) with confidence intervals, effect sizes, degrees of freedom and *P* value noted<br>*Give P values as exact values whenever suitable.*
[x]	[ ]	For Bayesian analysis, information on the choice of priors and Markov chain Monte Carlo settings
[x]	[ ]	For hierarchical and complex designs, identification of the appropriate level for tests and full reporting of outcomes
[x]	[ ]	Estimates of effect sizes (e.g. Cohen's *d*, Pearson's *r*), indicating how they were calculated

Our web collection on statistics for biologists contains articles on many of the points above.

Software and code

Policy information about availability of computer code

Data collection	Crystallography analysis was performed using: XDS (BUILT=20220220), AIMLESS (0.7.7), Phenix autobuild (1.19.2), COOT (0.9.6), Phenix Refine (1.19.2). Protein docking was performed using AutoDockVina (1.1.2) and ligand and receptor preparation was done in AutoDockTools (1.5.6). Phylogenetic tree was constructed in MEGAX (10.2.2). RNAseq reads related software are as follows (please see methods section for specific details): Trimmomatic (v0.32), Trinity (v20140717), Cutadapt (v3.4), Kallisto (v0.46.2).
Data analysis	Assay graphs were made in GraphPad Prism (8.2.0). Protein crystal structures/models were visualized in Pymol (2.3.2). Adobe Illustrator (15.0.0) was used to generate figures. Nucleic acid and protein sequences were analyzed/designed in Geneious Prime (2019.1.1). Protein sequence alignment was constructed in using ClustalW (v2.1). Chemical structures were generated in ChemDraw Professional (17.1) generally, and in MarvinSketch (19.19.0) for docking. ESPript3.0 online tool was used to generate the alignment figure. Protparam (ExPasy) online tool was used to calculate MW and extinction coefficients for determining protein concentrations.

For manuscripts utilizing custom algorithms or software that are central to the research but not yet described in published literature, software must be made available to editors and reviewers. We strongly encourage code deposition in a community repository (e.g. GitHub). See the Nature Portfolio guidelines for submitting code & software for further information.

Data

Policy information about availability of data

All manuscripts must include a data availability statement. This statement should provide the following information, where applicable:

- Accession codes, unique identifiers, or web links for publicly available datasets
- A description of any restrictions on data availability
- For clinical datasets or third party data, please ensure that the statement adheres to our policy

There are no restrictions on the availability of data. See the methods section in the main paper for details on the deposited transcriptome data and crystallographic data. Additional data are available from the corresponding author upon request.

Field-specific reporting

Please select the one below that is the best fit for your research. If you are not sure, read the appropriate sections before making your selection.

[x] Life sciences [ ] Behavioural & social sciences [ ] Ecological, evolutionary & environmental sciences

For a reference copy of the document with all sections, see nature.com/documents/nr-reporting-summary-flat.pdf

Life sciences study design

All studies must disclose on these points even when the disclosure is negative.

Sample size	Prior determination of sample size was not a consideration for our data. Replicates of 2-3 were used for enzyme assays, which is a standard number of replicates for these experiments.
Data exclusions	No data were excluded from the analyses.
Replication	The majority of the data presented in this study is representative of two or three experiments done in different days. Some mutagenesis data was performed once but all key variants that were discussed in the paper were repeated in follow-up experiments. All attempts at replication were successful.
Randomization	Randomization was not relevant for our study because replicates were done independently and on different days. Relicates were analyzed in no particular order.
Blinding	Blinding was not relevant for our study because group allocation was not necessary to collect and process data.

Reporting for specific materials, systems and methods

We require information from authors about some types of materials, experimental systems and methods used in many studies. Here, indicate whether each material, system or method listed is relevant to your study. If you are not sure if a list item applies to your research, read the appropriate section before selecting a response.

Materials & experimental systems

n/a	Involved in the study	
[x]	[ ]	Antibodies
[x]	[ ]	Eukaryotic cell lines
[x]	[ ]	Palaeontology and archaeology
[x]	[ ]	Animals and other organisms
[x]	[ ]	Human research participants
[x]	[ ]	Clinical data
[x]	[ ]	Dual use research of concern

Methods

n/a	Involved in the study	
[x]	[ ]	ChIP-seq
[x]	[ ]	Flow cytometry
[x]	[ ]	MRI-based neuroimaging
